# Inequalities in the prevalence of major depressive disorder in Brazilian slum populations: a cross-sectional analysis

**DOI:** 10.1017/S204579602100055X

**Published:** 2021-10-21

**Authors:** Charlie F. M. Pitcairn, Anthony A. Laverty, Jasper J. L. Chan, Oyinlola Oyebode, Matías Mrejen, Julia M. Pescarini, Daiane Borges Machado, Thomas V. Hone

**Affiliations:** 1 School of Public Health, Imperial College London, London, UK; 2 Department of Primary Care and Public Health, School of Public Health, Imperial College London, London, UK; 3 Warwick Medical School, University of Warwick, Coventry, UK; 4 São Paulo School of Business Administration, Fundação Getulio Vargas, São Paulo, Brazil; 5 Instituto de Estudos para Políticas de Saúde (IEPS), São Paulo, Brazil; 6 Centro de Integração de Dados e Conhecimentos para Saúde (Cidacs), Fundação Oswaldo Cruz, Salvador, Brazil; 7 Faculty of Epidemiology and Population Health, London School of Hygiene & Tropical Medicine, London, UK; 8 Center of Data and Knowledge Integration for Health, Instituto Gonçalo Moniz, Fundação Oswaldo Cruz, Salvador, Brazil; 9 Department of Global Health and Social Medicine, Harvard Medical School, Boston, Massachusetts, USA

**Keywords:** Depression, Brazil, slums, inequalities, PHQ-9, LMIC, urban health

## Abstract

**Aims:**

The mental health of slum residents is under-researched globally, and depression is a significant source of worldwide morbidity. Brazil's large slum-dwelling population is often considered part of a general urban-poor demographic. This study aims to identify the prevalence and distribution of depression in Brazil and compare mental health inequalities between slum and non-slum populations.

**Methods:**

Data were obtained from Brazil's 2019 National Health Survey. Slum residence was defined based on the UN-Habitat definition for slums and estimated from survey responses. Doctor-diagnosed depression, Patient Health Questionnaire (PHQ-9)-screened depression and presence of undiagnosed depression (PHQ-9-screened depression in the absence of a doctor's diagnosis) were analysed as primary outcomes, alongside depressive symptom severity as a secondary outcome. Prevalence estimates for all outcomes were calculated. Multivariable logistic regression models were used to investigate the association of socioeconomic characteristics, including slum residence, with primary outcomes. Depressive symptom severity was analysed using generalised ordinal logistic regression.

**Results:**

Nationally, the prevalence of doctor diagnosed, PHQ-9 screened and undiagnosed depression were 9.9% (95% confidence interval (CI): 9.5–10.3), 10.8% (95% CI: 10.4–11.2) and 6.9% (95% CI: 6.6–7.2), respectively. Slum residents exhibited lower levels of doctor-diagnosed depression than non-slum urban residents (8.6%; 95% CI: 7.9–9.3 *v.* 10.7%; 95% CI: 10.2–11.2), while reporting similar levels of PHQ-9-screened depression (11.3%; 95% CI: 10.4–12.1 *v.* 11.3%; 95% CI: 10.8–11.8). In adjusted regression models, slum residence was associated with a lower likelihood of doctor diagnosed (adjusted odds ratio (adjusted OR): 0.87; 95% CI: 0.77–0.97) and PHQ-9-screened depression (adjusted OR: 0.87; 95% CI: 0.78–0.97). Slum residents showed a greater likelihood of reporting less severe depressive symptoms. There were significant ethnic/racial disparities in the likelihood of reporting doctor-diagnosed depression. Black individuals were less likely to report doctor-diagnosed depression (adjusted OR: 0.66; 95% CI: 0.57–0.75) than white individuals. A similar pattern was observed in Mixed Black (adjusted OR: 0.72; 95% CI: 0.66–0.79) and other (adjusted OR: 0.63; 95% CI: 0.45–0.88) ethnic/racial groups. Slum residents self-reporting a diagnosis of one or more chronic non-communicable diseases had greater odds of exhibiting all three primary depression outcomes.

**Conclusions:**

Substantial inequalities characterise the distribution of depression in Brazil including in slum settings. People living in slums may have lower diagnosed rates of depression than non-slum urban residents. Understanding the mechanisms behind the discrepancy in depression diagnosis between slum and non-slum populations is important to inform health policy in Brazil, including in addressing potential gaps in access to mental healthcare.

## Introduction

Mental health morbidities, including major depressive disorder (depression), account for an ever-increasing proportion of the global disease burden (Liu *et al*., 2020). Depression is estimated to affect nearly 280 million people globally (Global Burden of Disease Collaborative Network, 2020). This burden is predominantly focused in low- and middle-income countries (LMICs) as over 80% of global depression-related disability comes from these settings (Global Burden of Disease Collaborative Network, 2020). Recognition of depression's role as a cause of disability and its association with deteriorating physical health (Patten *et al*., 2008) has been accompanied by action to raise the profile of mental health conditions at an international level. Target 3.4 of the United Nations (UN) Sustainable Development Goals (SDGs), aiming for a one-third reduction in premature mortality from non-communicable diseases (NCDs) by 2030 (United Nations Development Programme, 2021), includes tackling mental health challenges alongside those posed by other NCDs. Moreover, in 2019 the World Health Organization (WHO) launched a ‘Special Initiative for Mental Health’ (World Health Organization, 2019) aimed at expanding health coverage for common mental disorders, including depression, as a necessity for achieving Universal Health Coverage.

Brazil has a considerable and growing country-level burden of depression, increasing from 7.9% in 2013 to 10.8% in 2019 (Souza Lopes *et al*., 2021). Brazil's Unified Health System (Sistema Único de Saúde; SUS) has a strong focus on primary care and health promotional activities at the local level (Macinko and Harris, 2015). Teams of community health workers, nurses and physicians – under the national Family Health Strategy (FHS) – provide basic mental health services, frequently supported by mental health specialists (Soares and de Oliveira, 2016). Expansion of the FHS has been shown to reduce urban inequalities in health outcomes (Bastos *et al*., 2017; Pinto and Giovanella, 2018; Hone *et al*., 2020). Despite this research, the mental health of Brazil's slum populations remains infrequently studied.

This lack of research into the mental health of the more than 1 billion people estimated to live in slums (UN-Habitat, 2015) is a global issue. The health of slum residents is not only of importance due to the size of this population but also because of the impacts that slums, as diverse spatial entities and concentrations of deprivation (Rice and Rice, 2009; Nolan *et al*., 2018), have on mental health outcomes. Although no universally accepted definition of a slum has been formulated, they are broadly characterised as deprived urban areas with a lack of access to basic public services (Ezeh *et al*., 2017). Many slums exhibit common traits including violence, little open space for relaxation and poor sanitation (Lilford *et al*., 2017). Qualitative and cross-sectional quantitative research in India has found a considerable burden of common mental disorders, including anxiety and depression, in slum areas (Subbaraman *et al*., 2014). The impacts of lived environments on health are known as ‘neighbourhood effects’ and have been suggested to play a role in negatively mediating health outcomes in slum areas (Lilford *et al*., 2019). Studies conducted with people living in slums in Ghana found that community-level influences such as poor sanitation and crime can contribute to poor mental health outcomes (Greif and Nii-Amoo Dodoo, 2015), whereas studies from Hong Kong and other areas have shown that household-level deprivation (Cheung and Chou, 2019; Chung *et al*., 2020) and individual poor socioeconomic status (Lorant *et al*., 2007) are associated with increased rates of depressive symptoms.

Over 16% of Brazil's urban population are estimated to live in slums (The World Bank, 2018). Those living in slums are usually the largest and poorest urban population groups in Brazil and their mental health has been infrequently studied. Despite the distinction of slum areas in national statistics, Brazilian research on depression often examines only urban (Ferrari *et al*., 2013) and rural (Corrêa *et al*., 2020) differences, whereas the prevalence of depression within urban populations, including within slum populations have not been explored.

The lack of analysis of depression between different sociodemographic groups in Brazilian urban and slum environments provides an opportunity for further exploration. This study aimed to investigate how doctor diagnosed, screened and undiagnosed prevalence measures of depression vary between slum and non-slum populations and the Brazilian population at large. We also explored the socioeconomic patterns associated with the prevalence of these depression outcomes and establish whether they vary between slum and non-slum populations. Finally, we investigated how the severity of depressive symptoms varies in these same populations.

## Methods

### Study design

This observational, cross-sectional study made use of data collected during the 2019 Brazilian National Health Survey (Pesquisa Nacional de Saúde – PNS).

### Data source

The PNS is a nationwide household survey conducted by the Brazilian Institute of Geography and Statistics (IBGE) and the Brazilian Ministry of Health (Stopa *et al*., 2020). First carried out in 2013, it aims to ‘evaluate health conditions {and} health service access’ as well as perform ‘surveillance of non-communicable diseases and their social determinants’ (Stopa *et al*., 2020). The PNS targets individuals aged 15 and over, living in ‘permanent private dwellings’ (Stopa *et al*., 2020). In the PNS 2019, a total of 279 382 households and 94 114 respondents to the individual questionnaire were included. Individual respondents were randomly selected from all household members aged 15 and above. Data were collected between August 2019 and March 2020 (Stopa *et al*., 2020). The survey collected individual socio-demographic information (e.g. age, sex, ethnicity/race and education) and contained modules addressing such themes as: general health status, lifestyle, communicable and NCDs and health service usage, among others. Weighting of responses adjusted for likelihood of selection and rate of non-response by sex and age category.

### Ethical considerations

No ethical approval was required for this analysis as it uses secondary data. The individual anonymised dataset is publicly available from IBGE (https://www.ibge.gov.br/estatisticas/sociais/saude/9160-pesquisa-nacional-de-saude.html?=&t=downloads).

### Measures

#### Primary outcomes

This study considers three primary outcomes: PHQ-9 screened, doctor diagnosed and undiagnosed depression. The PNS used two metrics to gauge depression prevalence. It used the Patient Health Questionnaire-9 (PHQ-9) for depression screening alongside asking about previous depression diagnosis. PHQ-9-screened depression was determined by asking participants to respond to all nine questions from the PHQ-9 depression screening questionnaire. Doctor-diagnosed depression was established by asking respondents whether they had previously been given a diagnosis of depression by a psychiatrist or psychologist. Finally, we considered undiagnosed depression in individuals who met the PHQ-9 definition of depression but did not report previous diagnosis by a health professional.

Each of the PHQ-9 questionnaire's nine questions gathers information about one depressive symptom from the Diagnostic and Statistical Manual of Mental Disorders, Fifth Edition (DSM-V) (Kroenke *et al*., 2001). This study classified respondents as exhibiting symptoms indicative of significant major depressive disorder if their aggregate PHQ-9 score was ⩾10. Validation of the questionnaire in Brazil and elsewhere has found that this cut-off point confers considerable diagnostic validity (Spitzer *et al*., 1999; Kroenke *et al*., 2001; Manea *et al*., 2012; Santos *et al*., 2013) and maximises both sensitivity and specificity (Levis *et al*., 2019). Portuguese translations of PHQ-9 questions can be found in online Supplementary Table 1. Portuguese adaptations of the questionnaire have been performed by Brazilian psychiatrists in previously published research (Fraguas *et al*., 2006) which was used in Santos and colleagues' 2013 validation of the tool.

One further primary outcome, undiagnosed depression, was derived. A respondent was coded positively for undiagnosed depression if they met the PHQ-9 definition of depression but did not report previous diagnosis by a health professional.

#### Secondary outcomes

This study examined five categories of depressive symptom severity, defined by respondents' aggregate PHQ-9 score. Scores of 5–9, 10–14, 15–19 and ⩾20 were coded as mild, moderate, moderately-severe and severe depressive symptoms, respectively (Kroenke *et al*., 2001). Mild depressive symptoms (PHQ-9 score ⩽9) were not considered indicative of major depressive disorder for this analysis, as indicated above. However, even mild symptoms have been shown to negatively impact day-to-day mental wellbeing and quality of life (Coyne *et al*., 1994; Brenes, 2007) and thus were considered for analysis as a secondary outcome. A new categorical, ordinal variable, depression severity, was derived based off aggregate PHQ-9 score.

### Exposures of interest

The main exposure variable of interest was slum residence. A designation based on the operational definition of a slum of the UN Human Settlements Programme (UN-Habitat) (UN-Habitat, 2015) was used to classify respondents as slum or non-slum urban residents according to their responses to PNS questions: access – or lack thereof – to improved sanitation, water and household construction and the presence of overcrowded living conditions (⩾3 residents per room) (UN-Habitat, 2015). As no section of the PNS addressed security of respondents' residential tenure, that component of the UN-Habitat definition was not incorporated.

Urban-dwelling respondents who met one or more of these conditions were classified as slum residents. Available data were mapped onto the concepts contained in the UN-Habitat definition. Table 1 demonstrates the five characteristics, and the variables used to discern them, that were employed to categorise respondents as slum residents.

**Table 1.** Attributes used to define variable of interest (slum or non-slum)

Component of the UN-Habitat definition	Questions from PNS used identify presence/absence of component	Responses to questions indicative of component of slum definition
Urban setting (Ezeh *et al*., 2017)	Location of survey	Slums are an urban phenomenon Urban-dwelling respondents were considered for categorisation as slum residents
Overcrowding (UN-Habitat, 2015)	Number of rooms in household Number of household residents	A variable for overcrowding was derived and defined as 3 or more residents per habitable room
Lack of access to improved drinking water (UN-Habitat, 2015)	Main household water source Connection to water network (Y/N) Household access to piped water (Y/N)	Households drawing water from a ‘shallow water table or *cacimba*’, ‘source or spring’ or ‘other’ Households not connected to the general water distribution network Households to which water arrives non-piped
Lack of access to improved sanitation (UN-Habitat, 2015)	Presence of private bathroom for use of residents (Y/N) Uses a hole for excrement (Y/N) Destination of household drain Destination for household garbage	Households with 0 recorded private bathrooms/toilets Households who use a hole for excrement Households in which the bathroom drains into a ‘rudimentary pit; ditch; river, lake, stream or sea; other destination’
Lack of durable housing (UN-Habitat, 2015)	Material of household walls Material of household roof Material of household floor	Houses with walls made of ‘uncoated rammed earth’, ‘collected wood’. Roof made of ‘zinc, aluminium or sheet metal’. Floors made of ‘earth’

*Note*: Slum residents were defined as those who reported residing in an urban area and who met one or more of the other components of the modified UN-Habitat slum definition derived from PNS responses.

A range of other sociodemographic characteristics were included in the analyses. Selected variables were included either because of their previously determined association with depression in Brazil and other settings (e.g. age, sex, smoking status, alcohol consumption, comorbidity and physical activity) (Ford and Erlinger, 2004; Ströhle, 2009; Zivin *et al*., 2010; Stopa *et al*., 2015; Souza Lopes *et al*., 2016; Barros *et al*., 2017; Lever-van Milligen *et al*., 2017; Tampubolon and Maharani, 2017) or their potential to behave as confounders in the multivariable analysis of slum residence and depression prevalence (e.g. socioeconomic status, income and health service access) (Szwarcwald *et al*., 2011; Jankowska *et al*., 2012; Araya *et al*., 2018).

The specific sociodemographic attributes of respondents included were: sex (male or female); age (categorised into 15–24 years, 25–34, 35–44, 45–54, 55–64, 65–75, 75 and over); level of education (no formal education, incomplete elementary, complete elementary, incomplete secondary, complete secondary, incomplete tertiary, complete tertiary education); self-reported ethnicity/race (White, Black, Mixed Black, or other (Asian, Indigenous or not reported)); dwelling (urban slum, urban non-slum, rural); presence of comorbidities (categorised as having 1, 2 or 3 or more chronic conditions); registration with the Family Health Strategy (yes, no, unknown); physical activity history (some or no engagement in physical activity in the last 3 months); current smoking status (yes or no); alcohol consumption (currently drinking or not); enrolment in a private medical insurance scheme (PMI) (yes or no) and household income (reported as: less than ¼ minimum wage, ¼–½ min. wage, ½–1× min. wage, 1–2× min. wage, 2–3× min. wage, 3–5× min. wage, ⩾5× min. wage). The Brazilian annual minimum wage was USD 5198.40 in 2020 when adjusted for purchasing power (Organisation for Economic Co-operation and Development, 2021).

Respondents identifying as part of the Asian or Indigenous groups were combined with those who ignored the question and categorised as one subpopulation (other) for analysis due to their relatively small size compared to other categories. FHS registration status was included as a covariate due to the role it has been reported to play in reducing inequities in healthcare access in Brazil in the last 15 years (Paim *et al*., 2011; Hone *et al*., 2020).

### Statistical analyses

The prevalence of the outcomes was descriptively analysed. Frequencies were weighted to account for survey design and adjust for the composition of Brazil's adult population.

Multivariable binary logistic regression was performed to explore the association between dwelling (urban slum, urban non-slum, rural) and other socioeconomic factors on depression prevalence. This allowed for associations with individual covariates, including slum residence, to be examined after controlling for other explanatory variables. Covariates were tested for collinearity. All exhibited variance inflation factors of less than 2 (James *et al*., 2013) and were thus not excluded from the model. Adjusted odds ratios (adjusted OR) with 95% confidence intervals (95% CIs) and *p-*values were estimated. Statistical significance was defined as *p* < 0.05.

In subsequent analyses, dwelling was interacted with selected socio-demographic covariates to identify inequalities between slums and non-slum areas in the associations between depression outcomes and socioeconomic factors. This tested whether socioeconomic inequalities differed between slum and non-slum areas. Covariates selected for interaction analysis were: number of comorbidities, sex, age, education and ethnicity/race given their previously documented relationship to depression and healthcare access in Brazil (Stopa *et al*., 2015; Souza Lopes *et al*., 2016).

For secondary outcomes, generalised ordinal logistic (GOL) regression analysis was carried out to investigate the sociodemographic patterning of depressive symptom severity in Brazil. All covariates from the multivariable analysis were included. Brant testing of a normal ordinal logistic regression model revealed that the regression coefficients for each individual covariate differed significantly between each level of symptom severity. As such, the proportional odds and parallel line assumptions were violated, and a GOL model was selected (Williams, 2006).

All analyses were completed using Stata v16.1^®^ (Stata Corp., College Station, TX, USA).

## Results

### Descriptive analysis

A total of 90 846 individuals aged 15 years or over, who answered the individual questionnaire, were included in the analysis (Table 2). The proportion of individuals identifying as female was 53.0% (95% CI: 52.4–53.6). Most individuals were under age 45, with 18.6% (95% CI: 18.0–19.2) younger than 25. The proportion of individuals living in slums was 14.3% (95% CI: 13.7–15.0). The prevalence of doctor-diagnosed depression was 9.9% (95% CI: 9.5–10.3). The prevalence of PHQ-9-screened depression was higher at 10.8% (95% CI: 10.4–11.2). The proportion of undiagnosed depression was estimated at 6.9% (95% CI: 6.6–7.2).

**Table 2.** Description of the PNS sample that answered the individual health questionnaire (*n* = 90 846)

			Prevalence		
Respondents		Proportion of Brazilian population	Doctor-diagnosed depression	PHQ-9-screened depression (⩾10)	Undiagnosed depression
Total	90 846	100.0%	9.9% (9.5–10.3)	10.8% (10.4–11.2)	6.9% (6.6–7.2)
Sex					
Female	48 047	53.0% (52.4–53.6)	14.3% (13.7–14.9)	15.1% (14.4–15.7)	9.3% (8.8–9.8)
Male	42 799	47.1% (46.5–47.7)	4.9% (4.6–5.3)	6.0% (5.6–6.4)	4.2% (3.9–4.6)
Age category					
15–24	10 460	18.6% (18.0–19.2)	5.3% (4.6–6.2)	10.8% (9.7–11.9)	8.1% (7.2–9.1)
25–34	15 970	17.1% (16.6–17.6)	7.1% (6.4–7.8)	9.6% (8.8–10.5)	6.6% (5.9–7.3)
35–44	18 033	19.1% (18.7–19.6)	10.8% (9.9–11.7)	10.6% (9.8–11.3)	6.5% (6.0–7.2)
45–54	15 885	16.9% (16.4–17.3)	12.7% (11.8–13.7)	11.8% (10.9–12.7)	6.8% (6.1–7.5)
55–64	14 572	14.2% (13.8–14.6)	13.4% (12.3–14.5)	11.4% (10.5–12.4)	6.3% (5.6–7.0)
65–74	9965	8.9% (8.5–9.2)	11.8% (10.7–13.0)	10.0% (9.0–11.2)	6.2% (5.5–7.1)
75+	5961	5.2% (5.0–5.5)	10.2% (8.9–11.6)	12.0% (10.7–13.5)	8.6% (7.5–9.8)
Education level					
Without education	7658	5.8% (5.6–6.1)	8.0% (7.0–9.2)	12.8% (11.6–14.2)	9.1% (8.0–10.3)
Incomplete elementary or equivalent	28 618	28.5% (27.9–29.1)	11.0% (10.4–11.7)	12.1% (11.4–12.8)	7.7% (7.2–8.3)
Complete elementary or equivalent	7167	8.8% (8.5–9.1)	8.9% (7.8–10.2)	11.1% (9.9–12.5)	7.5% (6.5–8.7)
Incomplete secondary or equivalent	6353	8.6% (8.3–9.0)	7.7% (6.5–9.1)	11.3% (9.8–13.0)	7.3% (6.2–8.4)
Complete secondary or equivalent	23 471	28.4% (27.9–29.0)	8.6% (8.0–9.3)	9.4% (8.7–10.1)	6.2% (5.6–6.8)
Incomplete 3° or equivalent	3962	4.8% (4.6–5.1)	11.2% (9.3–13.4)	13.0% (10.9–15.3)	7.9% (6.4–9.8)
Graduated from 3°	13 617	15.0% (14.4–15.6)	12.2% (11.3–13.2)	9.0% (8.1–10.0)	5.0% (4.3–5.8)
Ethnicity/race					
White	33 133	42.9% (42.2–43.7)	12.1% (11.5–12.8)	10.6% (10.0–11.2)	6.3% (5.8–6.8)
Black	10 345	11.4% (11.0–11.8)	7.9% (7.1–8.8)	11.7% (10.7–12.8)	8.2% (7.4–9.1)
Mixed Black	45 994	44.2% (43.5–44.8)	8.3% (7.8–8.8)	10.7% (10.2–11.3)	7.2% (6.7–7.6)
Other	1374	1.5% (1.3–1.7)	7.7% (5.6–10.5)	10.0% (7.0–14.3)	7.6% (4.8–11.8)
Dwelling					
Urban slum	20 741	14.3% (13.7–15.0)	8.6% (7.9–9.3)	11.3% (10.4–12.1)	7.7% (7.0–8.4)
Urban non-slum	49 132	71.6% (70.8–72.4)	10.7% (10.2–11.2)	11.3% (10.8–11.8)	7.1% (6.8–7.5)
Rural	20 973	14.1% (13.7–14.5)	7.2% (6.6–7.8)	7.6% (6.9–8.2)	5.0% (4.5–5.5)
Number of comorbidities					
0	46 427	53.1% (52.5–53.8)	5.3% (5.0–5.7)	6.8% (6.3–7.2)	5.0% (4.6–5.4)
1	25 087	26.7% (26.2–27.2)	10.7% (10.0–11.4)	11.3% (10.6–12.1)	7.4% (6.8–8.0)
2	11 621	12.0% (11.6–12.3)	16.3% (15.1–17.6)	16.0% (14.9–17.3)	10.0% (9.1–11.0)
3+	7711	8.2% (7.9–8.6)	27.3% (25.3–29.3)	27.2% (25.6–29.0)	13.1% (11.9–14.4)
Registered with the FHS					
Registered	57 500	61.8% (60.7–62.9)	9.9% (9.4–10.3)	11.1% (10.6–11.7)	7.1% (6.8–7.5)
Not registered	22 512	26.9% (25.9–27.9)	10.0% (9.3–10.7)	10.5% (9.7–11.4)	6.8% (6.2–7.4)
Unknown	10 834	11.3% (10.8–11.9)	9.9% (8.8–11.0)	9.5% (8.5–10.6)	6.0% (5.2–6.9)
Physically active in last 3 months					
Yes	36 398	43.4% (42.7–44.1)	9.3% (8.6–9.9)	8.3% (7.8–8.9)	5.2% (4.8–5.6)
No	54 448	56.6% (55.9–57.3)	10.4% (9.9–10.9)	12.7% (12.1–13.2)	8.2% (7.8–8.7)
Smoking status					
Non-smoker	79 460	87.8% (87.5–88.2)	9.7% (9.3–10.1)	10.2% (9.8–10.7)	6.6% (6.3–6.9)
Smoker	11 386	12.2% (11.8–12.5)	11.4% (10.4–12.4)	14.7% (13.5–16.0)	9.3% (8.3–10.4)
Drinks alcohol					
No	55 430	58.9% (58.3–59.6)	11.1% (10.6–11.6)	11.9% (11.4–12.4)	7.4% (7.0–7.8)
Yes	35 416	41.1% (40.4–41.7)	8.2% (7.7–8.7)	9.2% (8.7–9.8)	6.2% (5.8–6.7)
Enrolled in private health plan					
Yes	20 568	26.6% (25.8–27.3)	12.4% (11.6–13.2)	9.3% (8.6–10.1)	5.3% (4.8–5.9)
No	70 278	73.4% (72.7–74.2)	9.0% (8.6–9.4)	11.3% (10.9–11.8)	7.5% (7.1–7.9)
Household income					
<0.25× MW	9550	8.2% (7.9–8.6)	8.1% (7.1–9.1)	12.7% (11.5–14.0)	9.0% (8.0–10.1)
0.25–0.5× MW	14 147	14.9% (14.4–15.3)	8.2% (7.4–9.0)	12.4% (11.4–13.4)	8.4% (7.6–9.3)
0.5–1.0× MW	26 406	29.2% (28.6–29.8)	9.3% (8.6–10.0)	11.5% (10.8–12.2)	7.6% (7.0–8.1)
1–2× MW	22 466	27.5% (27.0–28.1)	10.1% (9.4–10.9)	10.3% (9.5–11.1)	6.5% (5.9–7.2)
2–3× MW	7612	8.9% (8.6–9.3)	12.1% (10.6–13.7)	9.3% (8.1–10.7)	4.9% (4.2–5.7)
3–5× MW	5554	6.2% (5.9–6.5)	11.9% (10.6–13.4)	7.8% (6.6–9.2)	4.5% (3.7–5.4)
5+× MW	5089	5.0% (4.6–5.4)	13.7% (12.2–15.4)	7.9% (6.7–9.3)	4.0% (3.2–5.0)

FHS, family health strategy; MW, minimum wage.

*Note*: Prevalence of self-reported depression diagnosis, PHQ-9-screened depression and undiagnosed depression displayed (95% CI). PHQ-9 information was not available for 3268 of the 94 114 individual respondents.

**Fig. 1.**
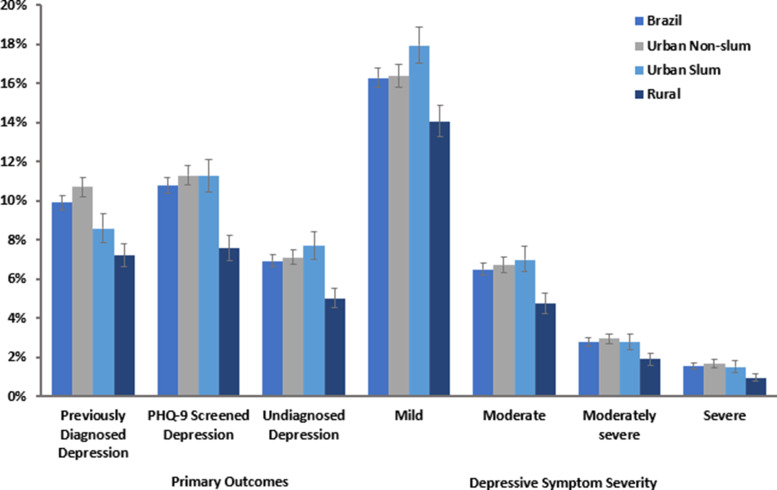
Estimated prevalence of depression outcomes (non-age-adjusted) in the Brazilian population by location of residence. *Note*: Error bars represent 95% confidence interval. Disease severity based on aggregate PHQ-9 score.

The prevalence of doctor-diagnosed depression was lower in urban slum-dwelling populations (8.6%; 95% CI: 7.9–9.3) than in urban non-slum populations (10.7%; 95% CI: 10.2–11.2) (Fig. 1). PHQ-9-diagnosed depression estimates were similar between the two groups, with slum and non-slum urban populations both reporting a prevalence of 11.3% with 95% CIs of 10.5–12.3 and 10.8–11.8, respectively. The prevalence of undiagnosed depression was similar in urban slum (7.7%; 95% CI: 7.0–8.4) and urban non-slum (7.1%; 95% CI: 6.8–7.5) subgroups but was lower in rural areas (5.0%; 95% CI: 4.5–5.5). Rural populations had a lower prevalence of all depression outcomes than urban populations. Aside from the higher prevalence of mild depression in urban-slum populations (17.9%; 95% CI: 17.0–19.0), there was no substantial difference in the distribution of symptom severity between the different urban sub-groups.

Females were more likely to report higher levels of all depression outcomes than males with the largest differences in doctor diagnosed (14.3%; 95% CI: 13.7–14.9 *v.* 4.9%; 95% CI: 4.6–5.3) and PHQ-9-screened depression (15.1%; 95% CI: 14.4–15.7 *v.* 6.0%; 95% CI: 5.6–6.4). The prevalence of doctor-diagnosed depression was found to increase with age from 5.3% (95% CI: 4.6–6.2) in 15–24-year-olds to a peak of 13.4% (95% CI: 12.3–14.5) among 55–64-year-olds. Such a steep gradient was not visible in PHQ-9-screened depression, with 10.8% (95% CI: 9.7–11.9) of 15–24-year-olds classified as depressed compared to 11.4% (95% CI: 10.5–12.4) of 55–64-year-olds.

Rates of doctor-diagnosed depression were lower in Black (7.9%; 95% CI: 7.1–8.8), and Mixed Black (8.3%; 95% CI: 7.8–8.8) ethnic/racial groups compared to those self-classifying as White (12.1%; 95% CI: 11.5–12.8). Rates of PHQ-9-diagnosed depression, however, were similar across racial/ethnic groups. Higher income groups reported greater rates of doctor-diagnosed depression (13.7%; 95% CI: 12.2–15.4 *v.* 8.1%; 95% CI: 7.1–9.1, highest *v.* lowest income category) and a lower prevalence of PHQ-9-screened depression (7.9%; 95% CI: 6.7–9.3 *v.* 12.7%: 95% CI: 11.5–14.0).

### Socioeconomic patterning of depression outcomes

In the multivariable logistic regression analysis we found that, after adjusting for all socioeconomic variables, individuals living in slums had a lower likelihood of reporting doctor diagnosed (adjusted OR: 0.87; 95% CI: 0.77–0.97) and PHQ-9-screened depression (adjusted OR: 0.87; 95% CI: 0.78–0.97) when compared to urban non-slum populations (Table 3).

**Table 3.** Results from multivariable logistic regression analysis

	Doctor-diagnosed depression		PHQ-9-screened depression		Undiagnosed depression	
	AOR	95% CI	AOR	95% CI	AOR	95% CI
Sex						
Male	1 (ref)	–	–	–	–	–
Female	2.77***	(2.52–3.05)	2.43***	(2.22–2.67)	2.12***	(1.90–2.37)
Age category						
15–24	1 (ref)	–	–	–	–	–
25–34	1.21	(0.99–1.47)	0.78**	(0.67–0.92)	0.73***	(0.61–0.87)
35–44	1.58***	(1.30–1.92)	0.70***	(0.60–0.82)	0.61***	(0.52–0.72)
45–54	1.47***	(1.20–1.79)	0.61***	(0.52–0.72)	0.52***	(0.44–0.63)
55–64	1.17	(0.95–1.43)	0.43***	(0.36–0.51)	0.39***	(0.32–0.48)
65–74	0.82	(0.65–1.02)	0.31***	(0.25–0.38)	0.35***	(0.28–0.43)
75+	0.63***	(0.49–0.81)	0.34***	(0.27–0.42)	0.44***	(0.35–0.56)
Education level						
Without education	1 (ref)	–	–	–	–	–
Incomplete elementary or equivalent	1.34***	(1.13–1.60)	0.87*	(0.75–1.00)	0.80**	(0.69–0.94)
Complete elementary or equivalent	1.23	(0.99–1.54)	0.85	(0.70–1.03)	0.80*	(0.65–0.99)
Incomplete high school or equivalent	1.37*	(1.06–1.78)	0.88	(0.70–1.10)	0.71**	(0.56–0.90)
Complete high school or equivalent	1.20	(0.99–1.45)	0.74***	(0.63–0.87)	0.68***	(0.57–0.81)
Incomplete 3° or equivalent	1.78***	(1.32–2.40)	1.18	(0.91–1.53)	0.94	(0.71–1.26)
Graduated from 3°	1.37**	(1.11–1.71)	0.87	(0.70–1.07)	0.73*	(0.56–0.96)
Ethnicity/race						
White	1 (ref)	–	–	–	–	–
Black	0.66***	(0.57–0.75)	1.00	(0.88–1.14)	1.15	(0.99–1.34)
Mixed Black	0.72***	(0.66–0.79)	0.94	(0.86–1.03)	1.03	(0.92–1.15)
Other	0.63**	(0.45–0.88)	0.87	(0.55–1.37)	1.10	(0.64–1.91)
Dwelling						
Urban non-slum	1 (ref)	–	–	–	–	–
Urban slum	0.87*	(0.77–0.97)	0.87*	(0.78–0.97)	0.92	(0.82–1.03)
Rural	0.81***	(0.73–0.91)	0.58***	(0.51–0.65)	0.57***	(0.50–0.65)
Number of comorbidities						
0	1 (ref)	–	–	–	–	–
1	2.08***	(1.87–2.32)	2.04***	(1.83–2.27)	1.76***	(1.55–1.99)
2	3.35***	(2.95–3.82)	3.41***	(3.02–3.85)	2.68***	(2.31–3.10)
3+	6.62***	(5.68–7.71)	7.14***	(6.28–8.13)	3.66***	(3.11–4.30)
Registered with the FHS						
Registered	1 (ref)	–	–	–	–	–
Not registered	0.89*	(0.80–0.99)	1.00	(0.90–1.12)	1.05	(0.93–1.19)
Unknown	1.00	(0.87–1.15)	0.94	(0.82–1.07)	0.94	(0.80–1.10)
Physically active in last 3 months						
Yes	1 (ref)	–	–	–	–	–
No	1.00	(0.91–1.11)	1.36***	(1.25–1.49)	1.40***	(1.26–1.56)
Smoking status						
Non-smoker	1 (ref)	–	–	–	–	–
Smoker	1.44***	(1.28–1.62)	1.66***	(1.48–1.86)	1.50***	(1.30–1.72)
Drinks alcohol						
No	1 (ref)	–	–	–	–	–
Yes	0.82***	(0.75–0.90)	0.93	(0.84–1.02)	1.03	(0.92–1.15)
Enrolled in private health plan						
No	1 (ref)	–	–	–	–	–
Yes	1.14*	(1.02–1.27)	0.87*	(0.77–0.99)	0.84*	(0.72–0.98)
Household income						
<0.25× MW	1 (ref)	–	–	–	–	–
0.25–0.5× MW	1.00	(0.84–1.20)	0.97	(0.83–1.12)	0.93	(0.78–1.10)
0.5–1.0× MW	1.03	(0.88–1.20)	0.87	(0.76–1.00)	0.84*	(0.71–0.98)
1–2× MW	1.08	(0.92–1.27)	0.82*	(0.70–0.96)	0.78*	(0.65–0.94)
2–3× MW	1.23	(0.98–1.56)	0.77*	(0.61–0.97)	0.63***	(0.50–0.80)
3–5× MW	1.23	(0.99–1.54)	0.68**	(0.53–0.88)	0.62***	(0.47–0.82)
5+× MW	1.41**	(1.09–1.82)	0.73*	(0.56–0.96)	0.60**	(0.43–0.84)

AOR, adjusted odds ratio (fully adjusted model); 95% CI, 95% confidence intervals; FHS, family health strategy; MW, minimum wage.

*p < 0.05, **p < 0.01, ***p < 0.001.

Other socioeconomic inequalities persisted in adjusted regression models. When looking at doctor-diagnosed depression, there was a greater likelihood of depression in females (adjusted OR: 2.77; 95% CI: 2.52–3.05); those aged 35–44 (adjusted OR: 1.58; 95% CI: 1.30–1.92) and 45–54 (adjusted OR: 1.47; 95% CI: 1.20–1.79); and those with one (adjusted OR: 2.08; 95% CI: 1.87–2.32), two (adjusted OR: 3.36; 95% CI: 2.95–3.82) and three or more (adjusted OR: 6.62; 95% CI: 5.69–7.71) comorbidities. PMI enrolment (adjusted OR: 1.14; 95% CI: 1.02–1.27) and a household income of 5+ times the minimum wage (adjusted OR: 1.41; 95% CI: 1.09–1.82) were also associated with an increased likelihood of reporting doctor-diagnosed depression. A lower likelihood of reporting this outcome was observed in those who were of Black (adjusted OR: 0.66; 95% CI: 0.57–0.75), Mixed Black (adjusted OR: 0.72; 95% CI: 0.66–0.79) or other (adjusted OR: 0.63; 95% CI: 0.45–0.88) ethnic/racial groups.

When looking at PHQ-9-screened depression, we observed a higher likelihood of depression in females (adjusted OR: 2.43; 95% CI: 2.22–2.67), those who had not engaged in physical activity in the 3 months preceding the survey (adjusted OR: 1.36; 95% CI: 1.25–1.49) and in smokers (adjusted OR: 1.66; 95% CI: 1.48–1.86). Lower odds of PHQ-9-screened depression was observed in older age categories, for example an adjusted OR of 0.43 (95% CI: 0.36–0.51) in 55–64-year-olds and an adjusted OR of 0.34 (95% CI: 0.27–0.42) in those aged 75 and older. Lower odds of PHQ-9-screened depression were also observed in those in possession of PMI (adjusted OR: 0.87; 95% CI: 0.77–0.99), urban-slum residents (adjusted OR: 0.87; 95% CI: 0.78–0.97) and members of higher household income categories, for example those in receipt of 1–2 times (adjusted OR: 0.82; 95% CI: 0.70–0.96) and 2–3 times (adjusted OR: 0.77; 95% CI: 0.61–0.97) the minimum wage.

The presence of undiagnosed depression was positively associated with female sex (adjusted OR: 2.12; 95% CI: 1.90–2.37), lack of physical activity (adjusted OR: 1.40; 95% CI: 1.26–1.56) and being a smoker (adjusted OR: 1.50; 95% CI: 1.30–1.72). A similar relationship was observed in people reporting one (adjusted OR: 1.76; 95% CI: 1.55–1.99), two (adjusted OR: 2.68; 95% CI: 2.31–3.10) and three or more (adjusted OR: 3.66; 95% CI: 3.11–4.30) comorbidities. Increased age, income and education levels as well as PMI enrolment were also associated with a lower likelihood of undiagnosed depression.

### Slum impact on depression severity

Depressive symptom severity was defined using aggregate PHQ-9 score. Slum residents were significantly less likely (adjusted OR: 0.86; 95% CI: 0.78–0.96) than non-slum urban residents to be classified as exhibiting moderate, moderately-severe or severe depressive symptoms *v.* no or mild symptoms (Table 4; online Supplementary Table 2). Slum residents were also less likely (adjusted OR: 0.77; 95% CI: 0.66–0.89) than non-slum urban populations to exhibit moderately-severe or severe symptoms *v.* no, mild or moderate symptoms of depression.

**Table 4.** Results of generalised ordinal logistic regression analysis of depression by symptom severity (PHQ-9 score)

	0 *v*. 1, 2, 3, 4		0 and 1 *v*. 2, 3, 4		0, 1, 2 *v*. 3 and 4		0, 1, 2, 3 *v*. 4	
	AOR	95% CI	AOR	95% CI	AOR	95% CI	AOR	95% CI
Urban non-slum	1 (ref)	–	–	–	–	–	–	–
Urban slum	0.99	(0.92–1.07)	0.86**	(0.78–0.96)	0.77***	(0.66–0.89)	0.75*	(0.58–0.96)
Rural	0.67***	(0.62–0.73)	0.57***	(0.51–0.64)	0.52***	(0.44–0.61)	0.46***	(0.36–0.60)

AOR, adjusted odds ratio (fully adjusted model); 95% CI, 95% confidence intervals; FHS, family health strategy.

*Note*: 0 = no depression; 1 = mild symptoms, 2 = moderate symptoms; 3 = moderately-severe symptoms, 4 = severe symptoms.

*p < 0.05, **p < 0.01, ***p < 0.001.

### Variation in primary outcomes by socioeconomic characteristics in slum populations

Table 5 shows the interaction between dwelling (urban slum, urban non-slum and rural) with the number of reported comorbidities in the multivariable model (the only significant interaction identified). Slum residents with one and three or more comorbidities had 1.34 (95% CI: 1.02–1.76) and 1.59 (95% CI: 1.16–2.19) greater adjusted odds, respectively, of reporting doctor-diagnosed depression than non-slum urban residents (online Supplementary Table 3). Slum residents with two comorbidities were also more likely to exhibit PHQ-9 screened (adjusted OR: 1.48; 95% CI: 1.12–1.96) and undiagnosed depression (adjusted OR: 1.51; 95% CI: 1.09–2.09). Nearly all other interactions tested were non-significant (online Supplementary Tables 4–7).

**Table 5.** Results from interactions between slum residency and number of comorbidities

	Doctor-diagnosed depression		PHQ-9-screened depression		Undiagnosed depression	
	AOR	95% CI	AOR	95% CI	AOR	95% CI
Dwelling						
Urban non-slum	1 (ref)	–	–	–	–	–
Urban slum	0.69***	(0.57–0.84)	0.76**	(0.64–0.91)	0.82	(0.67–1.00)
Rural	0.84	(0.69–1.03)	0.49***	(0.40–0.60)	0.45***	(0.36–0.57)
Number of comorbidities						
0	1 (ref)	–	–	–	–	–
1	2.04***	(1.79–2.33)	1.99***	(1.74–2.26)	1.70***	(1.45–1.99)
2	3.34***	(2.87–3.90)	3.08***	(2.66–3.56)	2.34***	(1.96–2.80)
3+	6.08***	(5.07–7.30)	6.75***	(5.81–7.84)	3.44***	(2.85–4.16)
*Interaction*						
Urban slum-dwelling × Number of comorbidities						
0	1 (ref)	–	–	–	–	–
1	1.34*	(1.02–1.76)	1.15	(0.89–1.48)	1.07	(0.80–1.42)
2	1.25	(0.93–1.67)	1.48**	(1.12–1.96)	1.51*	(1.09–2.09)
3	1.59**	(1.16–2.19)	1.13	(0.85–1.51)	1.20	(0.85–1.69)

AOR, adjusted odds ratio (fully adjusted model); 95% CI, 95% confidence intervals; FHS, family health strategy; MW, minimum wage.

*p < 0.05, **p < 0.01, ***p < 0.001.

## Discussion

Inequalities in the distribution of major depressive disorder in Brazil are stark, including among the country's substantial slum-dwelling population. Although this study found that over one in ten Brazilian individuals exhibited depression, those who were older, female and of White ethnic/racial group reported higher rates of diagnosed depression. Younger people and those with lower levels of education and household income were more likely to have undiagnosed depression. Slum residents had lower levels of doctor-diagnosed depression, a similar level of PHQ-9-screened depression and reported less severe depressive symptoms than non-slum urban residents. However, people who live in slums with comorbidities were at an increased risk of depression than non-slum urban comorbid individuals.

These findings indicate a higher prevalence of PHQ-9-screened depression (10.8%) than studies from 2013 (7.9%) (Souza Lopes *et al*., 2016), suggesting increases in recent years. Our estimate is also considerably higher than the WHO's own estimates for Brazil (5.8%) and Peru (6.4%) (Hernández-Vásquez *et al*., 2020). Depression prevalence in urban populations (11.3%) was also greater than in 2013 (8.1%) and other studies from Sao Paulo in 2008 (9.4%) (Andrade *et al*., 2012).

There was a notable burden of undiagnosed depression (6.9%), suggesting barriers to healthcare seeking behaviours and gaps in access to mental health services. This is lower than found by researchers in Canada and Japan, determining the rates of undiagnosed depression to be 10.9% (Farid *et al*., 2020) and 8.5% (Yamabe *et al*., 2019), respectively. However, it is greater than 5.0% found by Lotfaliany and colleagues' analysis of the WHO SAGE Wave 1 study of adults in six LMICs (China, Ghana, India, Mexico, Russia and South Africa) (Lotfaliany *et al*., 2018).

Inequalities in the distribution of doctor diagnosed and screened depression are stark. The findings echo those of earlier research in Brazil and internationally, which shows female sex, increased age, comorbidities, and smoking are associated with increased odds of depression (Wittayanukorn *et al*., 2014; Stopa *et al*., 2015; Souza Lopes *et al*., 2016; Abdi *et al*., 2021). Although research has not determined a definitive cause for higher rates of depression in women, previous analysis has suggested that higher Gross National Income (GNI) and shifting gender roles can influence the ratio of depression between males and females (Rai *et al*., 2013). This study was unable to infer the relationship between smoking status and comorbidity and the depression outcomes studied.

The finding that non-White ethnic/racial groups reported a lower likelihood of doctor-diagnosed depression is also concordant with 2013 data from Brazil (Stopa *et al*., 2015). This could be an indicative of a gap in access to mental health diagnostic services among Black and Mixed Black ethnic/racial groups. Such a gap has been observed in access to depression treatment (Souza Lopes *et al*., 2016) as well as prenatal and maternal health services (Matijasevich *et al*., 2008), breast cancer screening (Oliveira *et al*., 2011) and overall healthcare utilisation (Boccolini and de Souza Junior, 2016).

Depression results across slum and non-slum populations were mixed. In descriptive prevalence estimates, slum residents had lower levels of doctor-diagnosed depression than non-slum urban residents but had similar levels of PHQ-9-screened depression. However, in adjusted regression models, slum populations had lower odds of doctor-diagnosed and PHQ-9-screened depression compared to non-slum urban populations. These findings suggest that slum residence was associated with a lower likelihood for depression even after adjusting for socioeconomic and health service factors. This is notable as it refutes the *a priori* expectation that slum residence could negatively impact mental health outcomes independently of socioeconomic factors (Lilford *et al*., 2017; Lilford *et al*., 2019).

One possible explanation for the lower likelihood of depression in adjusted models for slum dwellers could be specific social and community aspects of slum-dwelling which are protective against depression. Social capital, including community engagement, social networks and trust, has been related to improved mental health outcomes (Berkman *et al*., 2000) and has been found to mitigate poor mental health in slum settings (Rabbani *et al*., 2018). Alternatively, there may be explanations from factors not controlled for in this analysis. Intergenerational co-habitation is one-example, with evidence from Europe and Asia showing it is negatively associated with depressive symptoms (Silverstein *et al*., 2006; Courtin and Avendano, 2016). Multigenerational dwelling may be inadvertently captured by overcrowding measures used in determining slum residence. Furthermore, high levels of depression have been reported in homeless populations (Perry and Craig, 2015), who would have been excluded from the PNS in slums and as such may have impacted our findings.

The findings from this study also showed that national-level inequalities in the prevalence of depression persist in slums. There were non-significant interactions between slum-residence and socioeconomic variables except for quantity of comorbidities. These results support the idea that slums are not homogenous populations, and efforts to tackle inequalities within slum populations are important. Comorbidities increase both greater medical costs and functional impairment (Moussavi *et al*., 2007; Kang *et al*., 2015), contributing to depression. Slum inhabitants with comorbidities may be at a greater risk of depression as they may incur greater healthcare costs (Buigut *et al*., 2015), need to continue working despite functional impairment (Niessen *et al*., 2018) or might forgo healthcare. The built environment of slums may further exacerbate poor quality of life for those with comorbidities by increasing barriers to healthcare access.

There are several limitations to the study. First, accurately identifying slum-dwelling populations is challenging. This was not easy using survey data, meaning that this study relied on a household-level definition of slum residence. Therefore, we were unable to account for the contiguous nature of slums (Snyder *et al*., 2014) that distinguish them from standalone deprived housing. Furthermore, the PNS sample only included respondents living in ‘permanent private dwellings’ (Stopa *et al*., 2020) and may have excluded those slum residents with more precarious living situations (e.g. people experiencing homelessness). Second, our study's cross-sectional nature precludes causal inference and there may be other unmeasured variables that explain the associations found. Self-reporting bias (Althubaiti, 2016) on the part of survey respondents may also underestimate the true burden of depression in Brazilian slums as well as the country at large.

Future research may benefit from adopting a geospatial approach to slum definition when examining depression outcomes in Brazil. Identifying favela or slum census tracts from the PNS 2019 survey would improve accuracy. City-level analyses using this approach have taken place in Rio de Janeiro (Szwarcwald *et al*., 2011; Snyder *et al*., 2014). Additionally, the UN-Habitat definition of slums does not disaggregate which and how many of its five components a slum resident is experiencing (Hacker *et al*., 2013). Subsequent studies, making use of satellite data with high-resolution remote-sensing capabilities and land-cover data, could monitor evolutions in slum size between decennial censuses (Mahabir *et al*., 2018).

There are important policy-relevant implications from this study. Notably, that socioeconomic inequalities in depression persist both within and outside slums. There is a need to recognise and tackle the wider socioeconomic determinants of poor health and depression. Although underdiagnosis (Rathod *et al*., 2017) and undertreatment (Lund *et al*., 2012) of common mental disorders remain prevalent globally, strengthening community-based mental healthcare operations and the use of lay-workers has proved effective at improving mental health outcomes in LMICs (Patel *et al*., 2008). Further efforts by the Brazilian government to tackle the causes of NCDs alongside mental health should focus on bolstering the capacity of local health teams to identify common mental disorders such as depression.

## Conclusion

Major depressive disorder unequally impacts a large share of the Brazilian population including slum residents. There are persisting socioeconomic inequalities in depression in Brazil, and undiagnosed depression remains a challenge. Slum populations may have lower diagnosed rates of depression than non-slum populations, potentially attributable to a lack of healthcare access, but understanding the mechanisms behind this are important for tackling the determinants of poor mental health, providing appropriate high-quality healthcare services, and making progress towards the SDGs for health and inequalities.

## Supplementary material

For supplementary material accompanying this paper visit http://dx.doi.org/10.1017/S204579602100055X. click here to view supplementary material

## Availability of data and materials

The PNS 2019 dataset can be obtained from the following web address: https://www.ibge.gov.br/estatisticas/sociais/saude/9160-pesquisa-nacional-de-saude.html?=&t=downloads.
