# Supplementary material for: Inequalities in the prevalence of major depressive disorder in Brazilian slum populations: a cross-sectional analysis
Source: Epidemiol Psychiatr Sci. 2021 Oct 21;30:e66. doi: 10.1017/S204579602100055X (PMC8546499; doi:10.1017/S204579602100055X)
Supplement: Supplementary file 1 [file S204579602100055Xsup001.docx]

Supplementary Table 1 – A sample PHQ-9 Questionnaire adapted from Kroenke et al., 2001 (Kroenke, Spitzer & Williams, 2001) demonstrating questions in English and Portuguese.

| **Questions** | | **Possible Answers (Score 0-3)** | | | |
| --- | --- | --- | --- | --- | --- |
| **Questions in English** | **Questions in Portuguese** | **Not at all** | **Several days** | **More than half the days** | **Nearly every day** |
| Over the last 2 weeks, how often have you had little interest or pleasure in doing things? | Nas duas últimas semanas, com que frequência o(a) Sr(a) teve pouco interesse ou não senitiu prazer em fazer as coisas? | **0** | **1** | **2** | **3** |
| Over the last 2 weeks, how often have you felt down, depressed, or hopeless? | Nas duas últimas semanas, com que frequência o(a) Sr(a) se sentiu deprimido(a), “pra baixo” ou sem perspectiva? | **0** | **1** | **2** | **3** |
| Over the last 2 weeks, how often have you had trouble falling or staying asleep, or sleeping too much? | Nas duas últimas semanas, com que frequência o(a) Sr(a) teve problemas no sono, como dificuldade para adormecer, acordar frequentemente à noite ou dormir mais do que de costume? | **0** | **1** | **2** | **3** |
| Over the last 2 weeks, how often have you felt tired or having little energy? | Nas duas últimas semanas, com que frequência o(a) Sr(a) teve problemas por não se sentir descansado(a) e disposto(a) durante o dia, sentindo-se cansado(a), sem ter energia? | **0** | **1** | **2** | **3** |
| Over the last 2 weeks, how often have you experienced poor appetite or overeating? | Nas duas últimas semanas, com que frequência o(a) Sr(a) teve problemas na alimentação, como ter falta de apetite ou comer muito mais do que de costume? | **0** | **1** | **2** | **3** |
| Over the last 2 weeks, how often have you felt bad about yourself—or that you are a failure or have let yourself or your family down? | Nas duas últimas semanas, com que frequência o(a) Sr(a) se sentiu mal consigo mesmo, se achando um fracasso ou achando que decepcionou sua família? | **0** | **1** | **2** | **3** |
| Over the last 2 weeks, how often have you had trouble concentrating on things, such as reading the newspaper or watching television? | Nas duas últimas semanas, com que frequência o(a) Sr(a) teve problemas para se concentrar nas suas atividades habituais? | **0** | **1** | **2** | **3** |
| Over the last 2 weeks, how often have you been bothered by moving or speaking so slowly that other people could have noticed? Or the opposite—being so fidgety or restless that you have been moving around a lot more than usual? | Nas duas últimas semanas, com que frequência o(a) Sr(a) teve lentidão para se movimentar ou falar, ou ao contrário ficou muito agitado(a) ou inquieto(a)? | **0** | **1** | **2** | **3** |
| Over the last 2 weeks, how often have you been bothered by thoughts that you would be better off dead or of hurting yourself in some way? | Nas duas últimas semanas, com que frequência o(a) Sr(a) pensou em se ferir de alguma maneira ou achou que seria melhor estar morto? | **0** | **1** | **2** | **3** |

Note: Questions in Portuguese are not direct translations but have been taken directly from the PNS 2019 questionnaire (Instituto Brasileiro de Geografia e Estatística, 2020).

Supplementary Table 2 - Results of generalized ordinal logistic regression analysis of depression by symptom severity (PHQ-9 score).

|  | **0 vs 1,2,3,4** | | **0 & 1 vs 2,3,4** | | **0,1,2 vs 3 & 4** | | **0,1,2,3 vs 4** | |
| --- | --- | --- | --- | --- | --- | --- | --- | --- |
|  | **AOR** | **95%CI** | **AOR** | **95%CI** | **AOR** | **95%CI** | **AOR** | **95%CI** |
| **Sex** |  |  |  |  |  |  |  |  |
| M | 1(ref) | - | - | - | - | - | - | - |
| F | 2.265*** | (2.134-2.405) | 2.458*** | (2.244-2.692) | 2.294*** | (2.000-2.631) | 2.306*** | (1.826-2.911) |
| **Age** |  |  |  |  |  |  |  |  |
| 15-24 | 1(ref) | - | - | - | - | - | - | - |
| 25 - 34 | 0.844*** | (0.764-0.932) | 0.778** | (0.666-0.908) | 0.888 | (0.707-1.114) | 0.976 | (0.651-1.464) |
| 35 - 44 | 0.759*** | (0.683-0.844) | 0.685*** | (0.588-0.798) | 0.877 | (0.694-1.108) | 0.827 | (0.552-1.239) |
| 45 - 54 | 0.653*** | (0.586-0.729) | 0.598*** | (0.511-0.699) | 0.704** | (0.560-0.885) | 0.678 | (0.440-1.046) |
| 55 - 64 | 0.487*** | (0.432-0.548) | 0.426*** | (0.359-0.505) | 0.542*** | (0.421-0.698) | 0.525** | (0.337-0.817) |
| 65 - 74 | 0.394*** | (0.347-0.448) | 0.300*** | (0.247-0.365) | 0.330*** | (0.247-0.440) | 0.312*** | (0.184-0.529) |
| 75 + | 0.394*** | (0.335-0.463) | 0.336*** | (0.271-0.416) | 0.402*** | (0.294-0.550) | 0.374*** | (0.215-0.649) |
| **Education** |  |  |  |  |  |  |  |  |
| Without education | 1(ref) | - | - | - | - | - | - | - |
| Incomplete elementary or equivalent | 0.818** | (0.726-0.922) | 0.857* | (0.746-0.984) | 0.845 | (0.688-1.037) | 1.007 | (0.713-1.422) |
| Complete elementary or equivalent | 0.726*** | (0.622-0.848) | 0.835 | (0.693-1.005) | 0.812 | (0.623-1.058) | 0.730 | (0.468-1.137) |
| Incomplete secondary or equivalent | 0.822* | (0.695-0.973) | 0.879 | (0.706-1.094) | 0.945 | (0.695-1.284) | 1.291 | (0.795-2.095) |
| Complete secondary or equivalent | 0.701*** | (0.611-0.804) | 0.730*** | (0.624-0.853) | 0.715** | (0.571-0.897) | 0.780 | (0.530-1.146) |
| Incomplete 3° or equivalent |  | (0.847-1.231) | 1.167 | (0.902-1.510) | 1.231 | (0.880-1.724) | 1.043 | (0.558-1.949) |
| Graduated from 3° | 0.827* | (0.706-0.970) | 0.856 | (0.698-1.048) | 0.807 | (0.604-1.079) | 0.924 | (0.541-1.577) |
| **Race** |  |  |  |  |  |  |  |  |
| White | 1(ref) | - | - | - | - | - | - | - |
| Black | 0.984 | (0.897-1.080) | 1.010 | (0.889-1.147) | 1.071 | (0.895-1.282) | 1.125 | (0.820-1.543) |
| Mixed Race | 0.967 | (0.907-1.031) | 0.943 | (0.865-1.028) | 0.968 | (0.841-1.113) | 0.883 | (0.698-1.117) |
| Other | 0.883 | (0.653-1.193) | 0.883 | (0.574-1.359) | 0.799 | (0.509-1.255) | 0.341** | (0.166-0.703) |
| **Dwelling** |  |  |  |  |  |  |  |  |
| Urban Non-slum | 1(ref) | - | - | - | - | - | - | - |
| Urban Slum | 0.991 | (0.920-1.068) | 0.864* | (0.779-0.959) | 0.767*** | (0.663-0.887) | 0.745* | (0.580-0.957) |
| Rural | 0.669*** | (0.616-0.725) | 0.573*** | (0.509-0.645) | 0.18*** | (0.438-0.613) | 0.460*** | (0.356-0.596) |
| **Comorbidities** |  |  |  |  |  |  |  |  |
| No comorbidities | 1(ref) | - | - | - | - | - | - | - |
| 1 | 1.906*** | (1.778-2.042) | 2.062*** | (1.856-2.291) | 2.386*** | (2.019-2.819) | 2.299*** | (1.711-3.089) |
| 2 | 3.151*** | (2.891-3.434) | 3.390*** | (3.005-3.825) | 3.843*** | (3.220-4.588) | 3.022*** | (2.219-4.116) |
| 3+ | 6.238*** | (5.621-6.922) | 7.075*** | (6.232-8.032) | 8.315*** | (6.887-10.040) | 7.899*** | (5.855-10.656) |
| **FHS registration** | | | | | | | | |
| Registered | 1(ref) | - | - | - | - | - | - | - |
| Not Registered | 0.994 | (0.922-1.072) | 1.003 | (0.902-1.115) | 0.965 | (0.835-1.116) | 0.869 | (0.694-1.088) |
| Unknown | 0.966 | (0.880-1.061) | 0.939 | (0.821-1.075) | 0.894 | (0.736-1.086) | 0.917 | (0.676-1.244) |
| **Physical Activity** | | | | | | | | |
| PA in last 3m | 1(ref) | - | - | - | - | - | - | - |
| No PA in last 3m | 1.181*** | (1.113-1.253) | 1.359*** | (1.243-1.485) | 1.510*** | (1.305-1.747) | 1.603** | (1.209-2.125) |
| **Tobacco use** |  |  |  |  |  |  |  |  |
| Non-smoker | 1(ref) | - | - | - | - | - | - | - |
| Smoker | 1.517*** | (1.401-1.643) | 1.670*** | (1.492-1.869) | 1.695*** | (1.452-1.978) | 1.932*** | (1.502-2.486) |
| **Alcohol use** |  |  |  |  |  |  |  |  |
| Non-drinker | 1(ref) | - | - | - | - | - | - | - |
| Drinker | 1.039 | (0.972-1.110) | 0.922 | (0.843-1.009) | 0.919 | (0.807-1.046) | 0.776* | (0.623-0.966) |
| **PMI Enrolment** | | |  |  |  |  |  |  |
| No | 1(ref) | - | - | - | - | - | - | - |
| Yes | 0.980 | (0.899-1.068) | 0.869* | (0.767-0.986) | 0.796* | (0.668-0.949) | 0.813 | (0.607-1.089) |
| **Income** |  |  |  |  |  |  |  |  |
| < 0.25 x MW | 1(ref) | - | - | - | - | - | - | - |
| 0.25 - 0.5 x MW | 0.965 | (0.860-1.083) | 0.985 | (0.851-1.139) | 1.022 | (0.833-1.254) | 0.930 | (0.663-1.304) |
| 0.5 - 1.0 x MW | 0.884* | (0.799-0.978) | 0.883 | (0.772-1.009) | 0.834 | (0.689-1.010) | 0.773 | (0.561-1.063) |
| 1-2 x MW | 0.791*** | (0.706-0.886) | 0.832* | (0.713-0.970) | 0.779* | (0.629-0.964) | 0.749 | (0.528-1.063) |
| 2-3 x MW | 0.762*** | (0.650-0.895) | 0.794* | (0.631-0.999) | 0.956 | (0.668-1.368) | 0.766 | (0.415-1.413) |
| 3-5 x MW | 0.728*** | (0.615-0.862) | 0.701** | (0.545-0.902) | 0.645* | (0.451-0.921) | 0.468* | (0.251-0.873) |
| 5+ x MW | 0.789* | (0.656-0.948) | 0.748* | (0.577-0.968) | 0.847 | (0.556-1.290) | 0.737 | (0.382-1.422) |
| **Constant** | 0.275*** | (0.226-0.335) | 0.076*** | (0.060-0.096) | 0.021*** | (0.015-0.030) | 0.008*** | (0.004-0.015) |

Note: 0 = No depression; 1 = Mild Symptoms, 2 = Moderate Symptoms; 3 = Moderately-severe Symptoms, 4 = Severe Symptoms. AOR = Adjusted odds ratio; 95%CI = 95% Confidence Intervals; FHS = Family Health Strategy; MW = Minimum Wage. *p<0.05, **p<001, ***p<0.001

Supplementary Table 3 – Complete results from interactions between slum residency and number of comorbidities.

|  | **Doctor Diagnosed Depression** | | | **PHQ-9 Screened Depression** | | | | **Undiagnosed Depression** | | |
| --- | --- | --- | --- | --- | --- | --- | --- | --- | --- | --- |
|  | **AOR** | **95%CI** | | **AOR** | | **95%CI** | | **AOR** | | **95%CI** |
| **Sex** |  |  | |  | |  | |  | |  |
| Male | 1 (ref) | - | | - | | - | | - | | - |
| Female | 2.771*** | (2.517-3.051) | | 2.428*** | | (2.212-2.665) | | 2.115*** | | (1.894-2.361) |
| **Age Category** |  |  | |  | |  | |  | |  |
| 15-24 | 1 (ref) | - | | - | | - | | - | | - |
| 25 - 34 | 1.204 | (0.985-1.472) | | 0.781** | | (0.666-0.915) | | 0.728*** | | (0.612-0.866) |
| 35 - 44 | 1.575*** | (1.297-1.912) | | 0.700*** | | (0.600-0.816) | | 0.610*** | | (0.516-0.721) |
| 45 - 54 | 1.464*** | (1.201-1.784) | | 0.613*** | | (0.523-0.718) | | 0.525*** | | (0.438-0.630) |
| 55 - 64 | 1.166 | (0.952-1.427) | | 0.434*** | | (0.364-0.516) | | 0.394*** | | (0.322-0.482) |
| 65 - 74 | 0.822 | (0.657-1.030) | | 0.310*** | | (0.254-0.378) | | 0.348*** | | (0.280-0.432) |
| 75 + | 0.634*** | (0.492-0.817) | | 0.339*** | | (0.272-0.422) | | 0.446*** | | (0.352-0.564) |
| **Education Level** |  |  | |  | |  | |  | |  |
| Without education | 1 (ref) | - | | - | | - | | - | | - |
| Incomplete elementary or equivalent | 1.359*** | (1.141-1.619) | | 0.883 | | (0.767-1.018) | | 0.820* | | (0.698-0.963) |
| Complete elementary or equivalent | 1.247 | (0.997-1.561) | | 0.871 | | (0.720-1.054) | | 0.820 | | (0.661-1.017) |
| Incomplete secondary or equivalent | 1.392* | (1.069-1.812) | | 0.901 | | (0.720-1.129) | | 0.731** | | (0.577-0.927) |
| Complete secondary or equivalent | 1.212 | (0.998-1.472) | | 0.754*** | | (0.642-0.886) | | 0.691*** | | (0.576-0.829) |
| Incomplete 3° or equivalent | 1.797*** | (1.330-2.428) | | 1.198 | | (0.920-1.559) | | 0.958 | | (0.718-1.278) |
| Graduated from 3° | 1.385** | (1.111-1.725) | | 0.882 | | (0.715-1.088) | | 0.747* | | (0.570-0.980) |
| **Ethnicity/Race** |  |  | |  | |  | |  | |  |
| White | 1 (ref) | - | | - | | - | | - | | - |
| Black | 0.655*** | (0.570-0.754) | | 0.997 | | (0.875-1.135) | | 1.152 | | (0.992-1.337) |
| Mixed Race | 0.724*** | (0.661-0.793) | | 0.943 | | (0.863-1.030) | | 1.028 | | (0.921-1.147) |
| Other | 0.628** | (0.450-0.877) | | 0.865 | | (0.549-1.364) | | 1.100 | | (0.636-1.904) |
| **Dwelling** |  |  | |  | |  | |  | |  |
| Urban Non-slum | 1 (ref) | - | | - | | - | | - | | - |
| Urban Slum | 0.687*** | (0.565-0.835) | | 0.764** | | (0.639-0.913) | | 0.816* | | (0.667-0.989) |
| Rural | 0.844 | (0.695-1.026) | | 0.491*** | | (0.404-0.596) | | 0.454*** | | (0.363-0.569) |
| **Number of comorbidities** |  |  | |  | |  | |  | |  |
| 0 | 1 (ref) | - | | - | | - | | - | | - |
| 1 | 2.042*** | (1.791-2.328) | | 1.986*** | | (1.742-2.265) | | 1.700*** | | (1.433-1.958) |
| 2 | 3.343*** | (2.867-3.899) | | 3.082*** | | (2.665-3.564) | | 2.344*** | | (1.960-2.803) |
| 3+ | 6.083*** | (5.070-7.299) | | 6.747*** | | (5.810-7.835) | | 3.442*** | | (2.851-4.156) |
| **Registered with the FHS** |  |  | |  | |  | |  | |  |
| Registered | 1 (ref) | - | | - | | - | | - | | - |
| Not Registered | 0.890* | (0.803-0.985) | | 1.002 | | (0.900-1.116) | | 1.052 | | (0.930-1.192) |
| Unknown | 0.997 | (0.864-1.150) | | 0.931 | | (0.812-1.066) | | 0.934 | | (0.799-1.092) |
| **Physically Active in last 3 months** |  |  | |  | |  | |  | |  |
| Yes | 1 (ref) | - | | - | | - | | - | | - |
| No | 1.004 | (0.909-1.109) | | 1.364*** | | (1.247-1.491) | | 1.400*** | | (1.260-1.557) |
| **Smoking Status** |  |  | |  | |  | |  | |  |
| Non-smoker | 1 (ref) | - | | - | | - | | - | | - |
| Smoker | 1.437*** | (1.277-1.617) | | 1.659*** | | (1.479-1.860) | | 1.494*** | | (1.296-1.722) |
| **Drinks Alcohol** |  |  | |  | |  | |  | |  |
| No | 1 (ref) | - | | - | | - | | - | | - |
| Yes | 0.821*** | (0.746-0.903) | | 0.927 | | (0.845-1.016) | | 1.028 | | (0.921-1.148) |
| **Enrolled in Private Health Plan** |  |  | |  | |  | |  | |  |
| No | 1 (ref) | - | | - | | - | | - | | - |
| Yes | 1.139* | (1.020-1.272) | | 0.875* | | (0.770-0.994) | | 0.838* | | (0.719-0.978) |
| **Household Income** |  |  | |  | |  | |  | |  |
| < 0.25 x MW | 1 (ref) | - | | - | | - | | - | | - |
| 0.25 - 0.5 x MW | 0.995 | (0.835-1.187) | | 0.956 | | (0.822-1.111) | | 0.917 | | (0.769-1.092) |
| 0.5 - 1.0 x MW | 1.018 | (0.870-1.193) | | 0.858* | | (0.745-0.988) | | 0.823* | | (0.698-0.971) |
| 1-2 x MW | 1.066 | (0.906-1.254) | | 0.804** | | (0.685-0.945) | | 0.771** | | (0.640-0.928) |
| 2-3 x MW | 1.224 | (0.970-1.544) | | 0.756* | | (0.598-0.956) | | 0.621*** | | (0.489-0.788) |
| 3-5 x MW | 1.222 | (0.979-1.527) | | 0.673** | | (0.521-0.869) | | 0.615*** | | (0.464-0.814) |
| 5+ x MW | 1.398** | (1.084-1.802) | | 0.724* | | (0.555-0.944) | | 0.594** | | (0.425-0.829) |
| **INTERACTIONS** |  |  | |  | |  | |  | |  |
| **Urban slum-dwelling x Number of comorbidities** | | |  | |  | |  | |  | |
| 0 | 1 (ref) | - | | - | | - | | - | | - |
| 1 | 1.344* | (1.024-1.764) | | 1.147 | | (0.891-1.476) | | 1.066 | | (0.800-1.419) |
| 2 | 1.247 | (0.932-1.670) | | 1.484** | | (1.122-1.963) | | 1.511* | | (1.093-2.090) |
| 3 | 1.592** | (1.156-2.194) | | 1.133 | | (0.851-1.508) | | 1.198 | | (0.852-1.686) |

Note: AOR = Adjusted odds ratio; 95%CI = 95% Confidence Intervals; FHS = Family Health Strategy, MW = Minimum wage; *p<0.05, **p<0.01, ***p<0.001

Supplementary Table 4 – Results of logistic regression with the dwelling variable interacted with sex variable.

|  | **Doctor Diagnosed Depression** | | **PHQ-9 Depression (>10)** | | **Undiagnosed Depression** | |
| --- | --- | --- | --- | --- | --- | --- |
|  | **AOR** | **95%CI** | **AOR** | **95%CI** | **AOR** | **95%CI** |
| **Sex** |  |  |  |  |  |  |
| Male |  |  |  |  |  |  |
| Female | 2.706*** | (2.413-3.036) | 2.365*** | (2.112-2.649) | 2.085*** | (1.823-2.385) |
| **Age Category** |  |  |  |  |  |  |
| 15-24 |  |  |  |  |  |  |
| 25 - 34 | 1.205 | (0.986-1.473) | 0.782** | (0.667-0.916) | 0.729*** | (0.613-0.868) |
| 35 - 44 | 1.579*** | (1.301-1.916) | 0.701*** | (0.602-0.818) | 0.611*** | (0.517-0.722) |
| 45 - 54 | 1.467*** | (1.204-1.787) | 0.613*** | (0.523-0.718) | 0.525*** | (0.437-0.630) |
| 55 - 64 | 1.168 | (0.954-1.429) | 0.432*** | (0.363-0.515) | 0.392*** | (0.321-0.480) |
| 65 - 74 | 0.819 | (0.654-1.025) | 0.308*** | (0.252-0.376) | 0.345*** | (0.278-0.430) |
| 75 + | 0.631*** | (0.489-0.813) | 0.337*** | (0.270-0.420) | 0.443*** | (0.350-0.561) |
| **Education Level** |  |  |  |  |  |  |
| Without education |  |  |  |  |  |  |
| Incomplete elementary or equivalent | 1.346*** | (1.132-1.600) | 0.866* | (0.751-0.997) | 0.803** | (0.685-0.943) |
| Complete elementary or equivalent | 1.230 | (0.985-1.535) | 0.849 | (0.702-1.028) | 0.799* | (0.644-0.992) |
| Incomplete secondary or equivalent | 1.373* | (1.058-1.782) | 0.877 | (0.700-1.099) | 0.713** | (0.562-0.904) |
| Complete secondary or equivalent | 1.196 | (0.986-1.451) | 0.736*** | (0.627-0.865) | 0.676*** | (0.563-0.810) |
| Incomplete 3° or equivalent | 1.776*** | (1.318-2.393) | 1.173 | (0.901-1.527) | 0.940 | (0.705-1.254) |
| Graduated from 3° | 1.373** | (1.105-1.707) | 0.865 | (0.701-1.066) | 0.733* | (0.559-0.960) |
| **Ethnicity/Race** |  |  |  |  |  |  |
| White |  |  |  |  |  |  |
| Black | 0.655*** | (0.569-0.754) | 0.998 | (0.877-1.136) | 1.154 | (0.994-1.339) |
| Mixed Race | 0.723*** | (0.660-0.792) | 0.943 | (0.864-1.030) | 1.028 | (0.922-1.147) |
| Other | 0.629** | (0.451-0.877) | 0.868 | (0.551-1.366) | 1.102 | (0.637-1.905) |
| **Dwelling** |  |  |  |  |  |  |
| Urban Non-slum |  |  |  |  |  |  |
| Urban Slum | 0.868 | (0.701-1.075) | 0.830 | (0.686-1.005) | 0.884 | (0.713-1.096) |
| Rural | 0.692*** | (0.578-0.829) | 0.511*** | (0.418-0.626) | 0.544*** | (0.429-0.689) |
| **Number of comorbidities** |  |  |  |  |  |  |
| 0 |  |  |  |  |  |  |
| 1 | 2.079*** | (1.864-2.319) | 2.037*** | (1.831-2.266) | 1.758*** | (1.551-1.993) |
| 2 | 3.352*** | (2.942-3.818) | 3.401*** | (3.012-3.840) | 2.676*** | (2.312-3.097) |
| 3+ | 6.610*** | (5.674-7.701) | 7.133*** | (6.268-8.118) | 3.654*** | (3.108-4.296) |
| **Registered with the FHS** |  |  |  |  |  |  |
| Registered |  |  |  |  |  |  |
| Not Registered | 0.891* | (0.804-0.986) | 1.001 | (0.899-1.115) | 1.052 | (0.929-1.192) |
| Unknown | 1.000 | (0.866-1.153) | 0.934 | (0.815-1.070) | 0.938 | (0.802-1.096) |
| **Physically Active in last 3 months** |  |  |  |  |  |  |
| Yes |  |  |  |  |  |  |
| No | 1.003 | (0.909-1.107) | 1.363*** | (1.246-1.490) | 1.399*** | (1.259-1.556) |
| **Smoking Status** |  |  |  |  |  |  |
| Non-smoker |  |  |  |  |  |  |
| Smoker | 1.441*** | (1.280-1.621) | 1.662*** | (1.482-1.865) | 1.495*** | (1.297-1.724) |
| **Drinks Alcohol** |  |  |  |  |  |  |
| No |  |  |  |  |  |  |
| Yes | 0.821*** | (0.746-0.903) | 0.927 | (0.845-1.016) | 1.028 | (0.921-1.148) |
| **Enrolled in Private Health Plan** |  |  |  |  |  |  |
| No |  |  |  |  |  |  |
| Yes | 1.141* | (1.022-1.275) | 0.872* | (0.768-0.992) | 0.836* | (0.716-0.975) |
| **Household Income** |  |  |  |  |  |  |
| Less than 0.25 MW |  |  |  |  |  |  |
| 0.25 - 0.5 MW | 1.003 | (0.842-1.195) | 0.968 | (0.833-1.124) | 0.927 | (0.779-1.104) |
| 0.5 - 1.0 MW | 1.029 | (0.880-1.203) | 0.872 | (0.758-1.003) | 0.836* | (0.709-0.985) |
| 1-2 MW | 1.077 | (0.916-1.267) | 0.817* | (0.696-0.960) | 0.782** | (0.649-0.942) |
| 2-3 MW | 1.234 | (0.978-1.555) | 0.766* | (0.606-0.968) | 0.628*** | (0.495-0.797) |
| 3-5 MW | 1.232 | (0.986-1.538) | 0.681** | (0.527-0.881) | 0.622*** | (0.469-0.823) |
| 5+ MW | 1.408** | (1.092-1.814) | 0.731* | (0.560-0.955) | 0.599** | (0.428-0.837) |
| **INTERACTIONS** |  |  |  |  |  |  |
| **Urban-slum dwelling x Female Sex** |  |  |  |  |  |  |
| Male | 1 (ref) | - | - | - | - | - |
| Female | 0.998 | (0.786-1.267) | 1.069 | (0.860-1.329) | 1.052 | (0.823-1.347) |
| **Rural dwelling x Female Sex** |  |  |  |  |  |  |
| Male | 1 (ref) | - | - | - | - | - |
| Female | 1.253* | (1.011-1.554) | 1.188 | (0.950-1.485) | 1.079 | (0.824-1.413) |

Note: AOR = Adjusted odds ratio; 95%CI = 95% Confidence Intervals; FHS = Family Health Strategy, MW = Minimum wage; *p<0.05, **p<0.01, ***p<0.001

Supplementary Table 5 – Results of logistic regression with the dwelling variable interacted with Age variable.

|  | **Doctor Diagnosed Depression** | | **PHQ-9 Depression (>10)** | | **Undiagnosed Depression** | |
| --- | --- | --- | --- | --- | --- | --- |
|  | **AOR** | **95%CI** | **AOR** | **95%CI** | **AOR** | **95%CI** |
| **Sex** |  |  |  |  |  |  |
| Male | 1 (ref) | - | - | - | - | - |
| Female | 2.777*** | (2.522-3.056) | 2.438*** | (2.222-2.676) | 2.124*** | (1.902-2.372) |
| **Age Category** |  |  |  |  |  |  |
| 15-24 | 1 (ref) | - | - | - | - | - |
| 25 - 34 | 1.074 | (0.844-1.366) | 0.788* | (0.647-0.960) | 0.752* | (0.605-0.935) |
| 35 - 44 | 1.460** | (1.160-1.838) | 0.679*** | (0.562-0.821) | 0.601*** | (0.489-0.740) |
| 45 - 54 | 1.278* | (1.013-1.611) | 0.583*** | (0.482-0.704) | 0.515*** | (0.413-0.641) |
| 55 - 64 | 1.067 | (0.846-1.346) | 0.397*** | (0.323-0.489) | 0.370*** | (0.290-0.471) |
| 65 - 74 | 0.716* | (0.555-0.924) | 0.273*** | (0.217-0.345) | 0.312*** | (0.241-0.404) |
| 75 + | 0.569*** | (0.427-0.758) | 0.317*** | (0.246-0.408) | 0.436*** | (0.331-0.574) |
| **Education Level** |  |  |  |  |  |  |
| Without education | 1 (ref) | - | - | - | - | - |
| Incomplete elementary or equivalent | 1.370*** | (1.151-1.630) | 0.901 | (0.782-1.038) | 0.831* | (0.707-0.977) |
| Complete elementary or equivalent | 1.269* | (1.014-1.587) | 0.895 | (0.739-1.084) | 0.835 | (0.672-1.038) |
| Incomplete secondary or equivalent | 1.412** | (1.086-1.837) | 0.920 | (0.733-1.154) | 0.740* | (0.584-0.938) |
| Complete secondary or equivalent | 1.228* | (1.012-1.490) | 0.771** | (0.656-0.906) | 0.702*** | (0.584-0.843) |
| Incomplete 3° or equivalent | 1.798*** | (1.332-2.426) | 1.214 | (0.932-1.581) | 0.967 | (0.724-1.292) |
| Graduated from 3° | 1.412** | (1.135-1.756) | 0.901 | (0.731-1.111) | 0.757* | (0.578-0.992) |
| **Ethnicity/Race** |  |  |  |  |  |  |
| White | 1 (ref) | - | - | - | - | - |
| Black | 0.654*** | (0.568-0.753) | 0.997 | (0.875-1.135) | 1.153 | (0.993-1.338) |
| Mixed Race | 0.724*** | (0.661-0.792) | 0.944 | (0.864-1.031) | 1.029 | (0.923-1.148) |
| Other | 0.621** | (0.445-0.869) | 0.868 | (0.549-1.372) | 1.107 | (0.638-1.920) |
| **Dwelling** |  |  |  |  |  |  |
| Urban Non-slum | 1 (ref) | - | - | - | - | - |
| Urban Slum | 0.572** | (0.390-0.840) | 0.823 | (0.630-1.074) | 0.969 | (0.728-1.290) |
| Rural | 0.522** | (0.336-0.812) | 0.416*** | (0.298-0.580) | 0.422*** | (0.293-0.608) |
| **Number of comorbidities** |  |  |  |  |  |  |
| 0 | 1 (ref) | - | - | - | - | - |
| 1 | 2.084*** | (1.869-2.323) | 2.044*** | (1.837-2.274) | 1.763*** | (1.555-1.998) |
| 2 | 3.359*** | (2.949-3.826) | 3.417*** | (3.025-3.860) | 2.685*** | (2.319-3.108) |
| 3+ | 6.628*** | (5.692-7.717) | 7.180*** | (6.309-8.171) | 3.676*** | (3.126-4.322) |
| **Registered with the FHS** |  |  |  |  |  |  |
| Registered | 1 (ref) | - | - | - | - | - |
| Not Registered | 0.889* | (0.803-0.985) | 1.001 | (0.898-1.115) | 1.053 | (0.929-1.192) |
| Unknown | 0.998 | (0.865-1.151) | 0.934 | (0.816-1.070) | 0.938 | (0.802-1.096) |
| **Physically Active in last 3 months** |  |  |  |  |  |  |
| Yes | 1 (ref) | - | - | - | - | - |
| No | 1.002 | (0.908-1.107) | 1.363*** | (1.246-1.490) | 1.397*** | (1.256-1.553) |
| **Smoking Status** |  |  |  |  |  |  |
| Non-smoker | 1 (ref) | - | - | - | - | - |
| Smoker | 1.434*** | (1.275-1.613) | 1.655*** | (1.476-1.855) | 1.491*** | (1.293-1.718) |
| **Drinks Alcohol** |  |  |  |  |  |  |
| No | 1 (ref) | - | - | - | - | - |
| Yes | 0.820*** | (0.745-0.902) | 0.926 | (0.845-1.016) | 1.027 | (0.920-1.147) |
| **Enrolled in Private Health Plan** |  |  |  |  |  |  |
| No | 1 (ref) | - | - | - | - | - |
| Yes | 1.142* | (1.022-1.275) | 0.873* | (0.768-0.992) | 0.837* | (0.717-0.977) |
| **Household Income** |  |  |  |  |  |  |
| Less than 0.25 MW | 1 (ref) | - | - | - | - | - |
| 0.25 - 0.5 MW | 0.990 | (0.830-1.180) | 0.949 | (0.816-1.104) | 0.913 | (0.766-1.089) |
| 0.5 - 1.0 MW | 1.007 | (0.860-1.178) | 0.844* | (0.732-0.973) | 0.813* | (0.688-0.961) |
| 1-2 MW | 1.055 | (0.897-1.240) | 0.794** | (0.675-0.933) | 0.763** | (0.632-0.921) |
| 2-3 MW | 1.209 | (0.959-1.524) | 0.747* | (0.590-0.945) | 0.615*** | (0.484-0.782) |
| 3-5 MW | 1.210 | (0.969-1.511) | 0.665** | (0.514-0.859) | 0.609*** | (0.460-0.806) |
| 5+ MW | 1.386* | (1.076-1.785) | 0.719* | (0.551-0.938) | 0.590** | (0.422-0.825) |
| **INTERACTIONS** |  |  |  |  |  |  |
| **Urban slum-dwelling x Age Category** |  |  |  |  |  |  |
| 15-24 | 1 (ref) | - | - | - | - | - |
| 25 - 34 | 1.557 | (0.977-2.480) | 0.863 | (0.608-1.224) | 0.828 | (0.566-1.212) |
| 35 - 44 | 1.507 | (0.960-2.366) | 1.016 | (0.721-1.434) | 0.896 | (0.607-1.323) |
| 45 - 54 | 1.746* | (1.128-2.702) | 1.069 | (0.771-1.481) | 0.877 | (0.605-1.270) |
| 55 - 64 | 1.485 | (0.943-2.340) | 1.256 | (0.890-1.774) | 0.978 | (0.662-1.443) |
| 65 - 74 | 1.682* | (1.005-2.813) | 1.378 | (0.885-2.146) | 1.382 | (0.861-2.220) |
| 75 + | 1.660 | (0.938-2.936) | 1.155 | (0.733-1.819) | 0.885 | (0.539-1.455) |
| **Rural dwelling x Age Category** |  |  |  |  |  |  |
| 15-24 | 1 (ref) | - | - | - | - | - |
| 25 - 34 | 1.779* | (1.068-2.962) | 1.142 | (0.761-1.714) | 0.994 | (0.627-1.574) |
| 35 - 44 | 1.291 | (0.783-2.128) | 1.330 | (0.908-1.947) | 1.415 | (0.926-2.163) |
| 45 - 54 | 1.893* | (1.160-3.089) | 1.506* | (1.014-2.236) | 1.525 | (0.976-2.382) |
| 55 - 64 | 1.513 | (0.919-2.491) | 1.707* | (1.131-2.575) | 1.913** | (1.197-3.058) |
| 65 - 74 | 2.047** | (1.188-3.527) | 2.142*** | (1.377-3.331) | 1.807* | (1.088-3.001) |
| 75 + | 1.589 | (0.888-2.843) | 1.671* | (1.015-2.751) | 1.557 | (0.890-2.723) |

Note: AOR = Adjusted odds ratio; 95%CI = 95% Confidence Intervals; FHS = Family Health Strategy, MW = Minimum wage; *p<0.05, **p<0.01, ***p<0.001

Supplementary Table 6 – Results of logistic regression with the dwelling variable interacted with Education variable.

|  | **Doctor Diagnosed Depression** | | **PHQ-9 Depression (>10)** | | **Undiagnosed Depression** | |
| --- | --- | --- | --- | --- | --- | --- |
|  | **AOR** | **95%CI** | **AOR** | **95%CI** | **AOR** | **95%CI** |
| **Sex** |  |  |  |  |  |  |
| Male | 1 (ref) | - | - | - | - | - |
| Female | 2.774*** | (2.520-3.054) | 2.437*** | (2.221-2.675) | 2.125*** | (1.903-2.374) |
| **Age Category** |  |  |  |  |  |  |
| 15-24 | 1.206 | (0.987-1.474) | - | - | - | - |
| 25 - 34 | 1.579*** | (1.301-1.917) | 0.780** | (0.666-0.914) | 0.728*** | (0.612-0.866) |
| 35 - 44 | 1.469*** | (1.205-1.789) | 0.698*** | (0.598-0.813) | 0.607*** | (0.514-0.718) |
| 45 - 54 | 1.169 | (0.956-1.430) | 0.610*** | (0.521-0.716) | 0.522*** | (0.435-0.627) |
| 55 - 64 | 0.820 | (0.656-1.026) | 0.430*** | (0.361-0.512) | 0.391*** | (0.319-0.478) |
| 65 - 74 | 0.632*** | (0.491-0.814) | 0.307*** | (0.252-0.375) | 0.345*** | (0.277-0.429) |
| 75 + | 1.206 | (0.987-1.474) | 0.337*** | (0.270-0.420) | 0.443*** | (0.350-0.561) |
| **Education Level** |  |  |  |  |  |  |
| Without education | 1 (ref) | - | - | - | - | - |
| Incomplete elementary or equivalent | 1.361* | (1.044-1.775) | 0.901 | (0.732-1.108) | 0.794* | (0.632-0.998) |
| Complete elementary or equivalent | 1.260 | (0.923-1.721) | 0.895 | (0.693-1.155) | 0.787 | (0.593-1.045) |
| Incomplete secondary or equivalent | 1.444* | (1.015-2.054) | 0.963 | (0.719-1.289) | 0.714* | (0.529-0.964) |
| Complete secondary or equivalent | 1.212 | (0.921-1.595) | 0.774* | (0.624-0.959) | 0.690** | (0.545-0.873) |
| Incomplete 3° or equivalent | 1.879*** | (1.291-2.735) | 1.272 | (0.925-1.751) | 0.957 | (0.678-1.351) |
| Graduated from 3° | 1.386* | (1.039-1.849) | 0.909 | (0.702-1.178) | 0.746 | (0.542-1.026) |
| **Ethnicity/Race** |  |  | 0.901 | (0.732-1.108) |  |  |
| White | 1 (ref) | - | - | - | - | - |
| Black | 0.656*** | (0.570-0.754) | 0.997 | (0.876-1.135) | 1.153 | (0.993-1.337) |
| Mixed Race | 0.724*** | (0.661-0.793) | 0.943 | (0.864-1.030) | 1.028 | (0.922-1.147) |
| Other | 0.630** | (0.451-0.879) | 0.864 | (0.547-1.365) | 1.098 | (0.635-1.898) |
| **Dwelling** |  |  |  |  |  |  |
| Urban Non-slum | 1 (ref) | - | - | - | - | - |
| Urban Slum | 1.070 | (0.703-1.628) | 0.920 | (0.662-1.279) | 0.735 | (0.525-1.030) |
| Rural | 0.727 | (0.517-1.023) | 0.666** | (0.508-0.874) | 0.697* | (0.515-0.943) |
| **Number of comorbidities** |  |  |  |  |  |  |
| 0 | 1 (ref) | - | - | - | - | - |
| 1 | 2.080*** | (1.866-2.320) | 2.039*** | (1.833-2.268) | 1.761*** | (1.554-1.996) |
| 2 | 3.350*** | (2.941-3.817) | 3.405*** | (3.015-3.846) | 2.685*** | (2.319-3.107) |
| 3+ | 6.625*** | (5.688-7.716) | 7.148*** | (6.280-8.136) | 3.656*** | (3.110-4.298) |
| **Registered with the FHS** |  |  |  |  |  |  |
| Registered | 1 (ref) | - | - | - | - | - |
| Not Registered | 0.891* | (0.804-0.987) | 1.001 | (0.898-1.114) | 1.051 | (0.928-1.191) |
| Unknown | 1.000 | (0.867-1.154) | 0.934 | (0.815-1.070) | 0.935 | (0.800-1.093) |
| **Physically Active in last 3 months** |  |  |  |  |  |  |
| Yes | 1 (ref) | - | - | - | - | - |
| No | 1.002 | (0.907-1.106) | 1.363*** | (1.246-1.490) | 1.400*** | (1.259-1.557) |
| **Smoking Status** |  |  |  |  |  |  |
| Non-smoker | 1 (ref) | - | - | - | - | - |
| Smoker | 1.438*** | (1.278-1.619) | 1.658*** | (1.478-1.859) | 1.493*** | (1.295-1.721) |
| **Drinks Alcohol** |  |  |  |  |  |  |
| No | 1 (ref) | - | - | - | - | - |
| Yes | 0.820*** | (0.746-0.902) | 0.926 | (0.844-1.015) | 1.026 | (0.920-1.146) |
| **Enrolled in Private Health Plan** |  |  |  |  |  |  |
| No | 1 (ref) | - | - | - | - | - |
| Yes | 1.142* | (1.023-1.276) | 0.871* | (0.766-0.990) | 0.832* | (0.712-0.972) |
| **Household Income** |  |  |  |  |  |  |
| Less than 0.25 MW | 1 (ref) | - | - | - | - | - |
| 0.25 - 0.5 MW | 1.002 | (0.841-1.195) | 0.967 | (0.833-1.124) | 0.929 | (0.780-1.107) |
| 0.5 - 1.0 MW | 1.028 | (0.879-1.202) | 0.870 | (0.755-1.001) | 0.835* | (0.708-0.985) |
| 1-2 MW | 1.076 | (0.915-1.267) | 0.815* | (0.693-0.959) | 0.782* | (0.648-0.943) |
| 2-3 MW | 1.231 | (0.976-1.552) | 0.762* | (0.603-0.964) | 0.627*** | (0.494-0.796) |
| 3-5 MW | 1.230 | (0.985-1.535) | 0.678** | (0.525-0.877) | 0.620*** | (0.468-0.821) |
| 5+ MW | 1.410** | (1.094-1.817) | 0.731* | (0.560-0.954) | 0.597** | (0.427-0.836) |
| **INTERACTIONS** |  |  |  |  |  |  |
| **Urban slum-dwelling x Education Level** |  |  |  |  |  |  |
| Without education | 1 (ref) | - | - | - | - | - |
| Incomplete elementary or equivalent | 0.834 | (0.530-1.311) | 0.972 | (0.678-1.393) | 1.283 | (0.883-1.863) |
| Complete elementary or equivalent | 0.738 | (0.422-1.291) | 0.953 | (0.603-1.506) | 1.356 | (0.827-2.225) |
| Incomplete secondary or equivalent | 0.771 | (0.429-1.386) | 0.830 | (0.494-1.394) | 1.388 | (0.796-2.420) |
| Complete secondary or equivalent | 0.777 | (0.484-1.249) | 0.985 | (0.678-1.429) | 1.234 | (0.828-1.839) |
| Incomplete 3° or equivalent | 0.605 | (0.332-1.102) | 0.878 | (0.512-1.506) | 1.385 | (0.779-2.460) |
| Graduated from 3° | 0.845 | (0.502-1.420) | 0.870 | (0.534-1.418) | 1.074 | (0.648-1.779) |
| **Rural dwelling x Education Level** |  |  |  |  |  |  |
| Without education | 1 (ref) | - | - | - | - | - |
| Incomplete elementary or equivalent | 1.119 | (0.767-1.633) | 0.888 | (0.650-1.213) | 0.871 | (0.613-1.237) |
| Complete elementary or equivalent | 1.190 | (0.746-1.899) | 0.839 | (0.495-1.421) | 0.854 | (0.454-1.607) |
| Incomplete secondary or equivalent | 0.854 | (0.492-1.484) | 0.684 | (0.415-1.127) | 0.654 | (0.352-1.215) |
| Complete secondary or equivalent | 1.303 | (0.859-1.976) | 0.822 | (0.567-1.193) | 0.641* | (0.413-0.995) |
| Incomplete 3° or equivalent | 0.695 | (0.326-1.486) | 0.331** | (0.154-0.712) | 0.253** | (0.104-0.613) |
| Graduated from 3° | 1.385 | (0.842-2.280) | 1.222 | (0.773-1.929) | 0.926 | (0.540-1.588) |

Note: AOR = Adjusted odds ratio; 95%CI = 95% Confidence Intervals; FHS = Family Health Strategy, MW = Minimum wage; *p<0.05, **p<0.01, ***p<0.001

Supplementary Table 7 – Results of logistic regression with the dwelling variable interacted with Race/Ethnicity variable.

|  | **Doctor Diagnosed Depression** | | **PHQ-9 Depression (>10)** | | **Undiagnosed Depression** | |
| --- | --- | --- | --- | --- | --- | --- |
|  | **AOR** | **95%CI** | **AOR** | **95%CI** | **AOR** | **95%CI** |
| **Sex** |  |  |  |  |  |  |
| Male | 1 (ref) | - | - | - | - | - |
| Female | 2.776*** | (2.521-3.056) | 2.435*** | (2.218-2.672) | 2.120*** | (1.899-2.368) |
| **Age Category** |  |  |  |  |  |  |
| 15-24 | 1 (ref) | - | - | - | - | - |
| 25 - 34 | 1.201 | (0.983-1.469) | 0.780** | (0.666-0.914) | 0.729*** | (0.613-0.868) |
| 35 - 44 | 1.571*** | (1.295-1.906) | 0.699*** | (0.600-0.815) | 0.611*** | (0.517-0.722) |
| 45 - 54 | 1.456*** | (1.195-1.774) | 0.611*** | (0.521-0.716) | 0.526*** | (0.438-0.630) |
| 55 - 64 | 1.165 | (0.952-1.425) | 0.431*** | (0.362-0.514) | 0.392*** | (0.320-0.479) |
| 65 - 74 | 0.817 | (0.653-1.023) | 0.308*** | (0.252-0.376) | 0.346*** | (0.278-0.430) |
| 75 + | 0.629*** | (0.488-0.810) | 0.337*** | (0.270-0.420) | 0.443*** | (0.350-0.561) |
| **Education Level** |  |  |  |  |  |  |
| Without education | 1 (ref) | - | - | - | - | - |
| Incomplete elementary or equivalent | 1.337*** | (1.125-1.588) | 0.865* | (0.751-0.996) | 0.806** | (0.687-0.946) |
| Complete elementary or equivalent | 1.220 | (0.977-1.523) | 0.849 | (0.701-1.028) | 0.802* | (0.646-0.996) |
| Incomplete secondary or equivalent | 1.365* | (1.052-1.771) | 0.878 | (0.701-1.100) | 0.715** | (0.564-0.907) |
| Complete secondary or equivalent | 1.191 | (0.983-1.444) | 0.738*** | (0.628-0.867) | 0.679*** | (0.566-0.814) |
| Incomplete 3° or equivalent | 1.773*** | (1.315-2.390) | 1.176 | (0.904-1.532) | 0.944 | (0.708-1.259) |
| Graduated from 3° | 1.372** | (1.104-1.705) | 0.867 | (0.703-1.069) | 0.736* | (0.562-0.963) |
| **Ethnicity/Race** |  |  |  |  |  |  |
| White | 1 (ref) | - | - | - | - | - |
| Black | 0.642*** | (0.542-0.761) | 1.004 | (0.861-1.170) | 1.160 | (0.972-1.385) |
| Mixed Race | 0.763*** | (0.685-0.851) | 0.965 | (0.868-1.073) | 1.022 | (0.894-1.168) |
| Other | 0.550** | (0.365-0.830) | 0.794 | (0.448-1.408) | 1.109 | (0.575-2.138) |
| **Dwelling** |  |  |  |  |  |  |
| Urban Non-slum | 1 (ref) | - | - | - | - | - |
| Urban Slum | 0.891 | (0.744-1.067) | 0.898 | (0.749-1.077) | 0.933 | (0.754-1.155) |
| Rural | 0.940 | (0.799-1.105) | 0.614*** | (0.500-0.754) | 0.545*** | (0.433-0.686) |
| **Number of comorbidities** |  |  |  |  |  |  |
| 0 | 1 (ref) | - | - | - | - | - |
| 1 | 2.082*** | (1.868-2.322) | 2.042*** | (1.836-2.271) | 1.760*** | (1.553-1.995) |
| 2 | 3.355*** | (2.945-3.822) | 3.404*** | (3.014-3.843) | 2.675*** | (2.311-3.097) |
| 3+ | 6.607*** | (5.672-7.695) | 7.143*** | (6.276-8.129) | 3.661*** | (3.114-4.305) |
| **Registered with the FHS** |  |  |  |  |  |  |
| Registered | 1 (ref) | - | - | - | - | - |
| Not Registered | 0.891* | (0.805-0.987) | 1.001 | (0.898-1.115) | 1.052 | (0.928-1.192) |
| Unknown | 1.002 | (0.868-1.156) | 0.936 | (0.816-1.072) | 0.938 | (0.803-1.097) |
| **Physically Active in last 3 months** |  |  |  |  |  |  |
| Yes | 1 (ref) | - | - | - | - | - |
| No | 1.003 | (0.909-1.107) | 1.363*** | (1.247-1.491) | 1.399*** | (1.259-1.556) |
| **Smoking Status** |  |  |  |  |  |  |
| Non-smoker | 1 (ref) | - | - | - | - | - |
| Smoker | 1.442*** | (1.281-1.623) | 1.663*** | (1.482-1.865) | 1.495*** | (1.296-1.723) |
| **Drinks Alcohol** |  |  |  |  |  |  |
| No | 1 (ref) | - | - | - | - | - |
| Yes | 0.820*** | (0.745-0.902) | 0.926 | (0.845-1.015) | 1.027 | (0.920-1.146) |
| **Enrolled in Private Health Plan** |  |  |  |  |  |  |
| No | 1 (ref) | - | - | - | - | - |
| Yes | 1.143* | (1.023-1.277) | 0.873* | (0.768-0.992) | 0.835* | (0.715-0.975) |
| **Household Income** |  |  |  |  |  |  |
| Less than 0.25 MW | 1 (ref) | - | - | - | - | - |
| 0.25 - 0.5 MW | 0.998 | (0.838-1.189) | 0.868* | (0.755-0.999) | 0.927 | (0.778-1.105) |
| 0.5 - 1.0 MW | 1.021 | (0.873-1.193) | 0.814* | (0.694-0.956) | 0.836* | (0.709-0.987) |
| 1-2 MW | 1.068 | (0.908-1.256) | 0.764* | (0.605-0.964) | 0.782* | (0.649-0.944) |
| 2-3 MW | 1.226 | (0.973-1.544) | 0.682** | (0.528-0.880) | 0.628*** | (0.495-0.797) |
| 3-5 MW | 1.230 | (0.985-1.535) | 0.733* | (0.562-0.956) | 0.622*** | (0.470-0.823) |
| 5+ MW | 1.409** | (1.094-1.815) | 0.868* | (0.755-0.999) | 0.599** | (0.429-0.838) |
| **INTERACTIONS** |  |  |  |  |  |  |
| **Urban slum-dwelling x Race** |  |  |  |  |  |  |
| White | 1 (ref) | - | - | - | - | - |
| Black | 1.298 | (0.905-1.863) | 0.941 | (0.690-1.284) | 0.803 | (0.572-1.128) |
| Mixed Race | 0.856 | (0.678-1.082) | 0.948 | (0.761-1.182) | 1.030 | (0.792-1.340) |
| Other | 2.317* | (1.077-4.985) | 1.312 | (0.569-3.025) | 0.523 | (0.197-1.389) |
| **Rural dwelling x Race** |  |  |  |  |  |  |
| White | 1 (ref) | - | - | - | - | - |
| Black | 0.790 | (0.535-1.165) | 1.010 | (0.679-1.502) | 1.296 | (0.844-1.988) |
| Mixed Race | 0.766* | (0.620-0.947) | 0.871 | (0.680-1.115) | 1.011 | (0.762-1.341) |
| Other | 1.061 | (0.391-2.877) | 2.007 | (0.862-4.674) | 2.055 | (0.808-5.232) |

Note: AOR = Adjusted odds ratio; 95%CI = 95% Confidence Intervals; FHS = Family Health Strategy, MW = Minimum wage; *p<0.05, **p<0.01, ***p<0.001

**SUPPLEMENTARY REFERENCES**

**Instituto Brasileiro de Geografia e Estatística.** (2020) *Pesquisa Nacional de Saúde - PNS.* Instituto Brasileiro de Geografia e Estatística.

**Kroenke, K., Spitzer, R. L. & Williams, J. B**. (2001) The PHQ-9: validity of a brief depression severity measure. *Journal of General Internal Medicine.* 16 (9), 606-613. Available from: doi: 10.1046/j.1525-1497.2001.016009606.x. [Accessed Feb 15, 2021].

stylefix
